# Cross-dialectal Arabic translation: comparative analysis on large language models

**DOI:** 10.3389/frai.2025.1661789

**Published:** 2025-09-18

**Authors:** Ayah Beidas, Kousar Mohi, Fatme Ghaddar, Imtiaz Ahmad, Sa'Ed Abed

**Affiliations:** Computer Engineering Department, College of Engineering and Petroleum, Kuwait University, Kuwait City, Kuwait

**Keywords:** language models, GPT 3.5, GPT 4, GPT 5, Bard (Gemini), Arabic language, dialects

## Abstract

**Introduction:**

Exploring Arabic dialects in Natural Language Processing (NLP) is essential to understand linguistic variation and meet regional communication demands. Recent advances in Large Language Models (LLMs) have opened up new vistas for multilingual communication and text generation.

**Methods:**

This paper investigates the performance of GPT-3.5, GPT-4, and Bard (Gemini) on the QADI and MADAR datasets, while GPT-5 was evaluated exclusively on MADAR encompassing over 15 different countries. Several metrics have been used in the evaluation, such as cosine similarity, universal similarity encoder, sentence BERT, TER, ROUGE, and BLEU. In this study, different prompting techniques were used: zero-shot and few-shot. Zero-shot was employed for all dialects, and few-shot was employed only for the least translation performance dialect, Tunisian.

**Results:**

Analysis revealed that in the QADI dataset, GPT-4 significantly outperformed others in translating MSA to DA, with ANOVA tests showing strong significance (*p* < 0.05) in most metrics, except for BLEU and TER where it does not show significance, indicating comparable translation performance among models. Furthermore, GPT-4 was highest in semantic similarity compared to GPT-3.5 and Bard (Gemini), 0.66, 0.61, and 0.63, respectively. GPT-4 was the best in identifying overlapping sentences (i.e., those where the source and target are identical) with a combined average of 0.41 in BLEU and ROUGE-L. All LLMs scored TER values between 6% and 25%, indicating generally good translation quality. However, GPT models, especially GPT-5, responded better to prompting and translation to Levant countries compared to Bard (Gemini). For the MADAR dataset, no significant translation differences were observed in sentence-BERT, ROUGE-L, and TER, while differences are identified in cosine similarity, BLEU, and universal similarity encoder metrics. Therefore, GPT-5 is the top performer in identifying sentence overlaps measured by BLEU and ROUGE-L (combined average 0.37).

**Discussion:**

The few-shot approach did not show a significant improvement in translation performance, especially for GPT-4 and Bard (Gemini), while GPT-3.5 performed consistently. Zero-shot prompts were effective across dialects, while few-shot prompting, applied to the weakest-performing dialect (Tunisian), did not yield improvement. GPT-4 and Bard performed worse under this set-up, while GPT-3.5 remained consistent.

## 1 Introduction

In recent years, new horizons for multilingual communication, translation tasks, and text generation have been widely witnessed due to the advances made in large language models (LLMs) (Shaikh et al., 2023). Models such as GPT, developed by OpenAI and Google Bard (Gemini), have shown promising developments in this field (Kasneci et al., 2023). Such models have demonstrated outstanding skills in handling diverse languages and dialects with the influential role of deep learning techniques and the processing of massive volumes of textual data. According to studies conducted in 2019 by Ethnologue (Eberhard et al., 2019), the total number of dialects spoken around the globe is expected to be 7,111, where a majority of these dialects are found on the Internet through platforms such as Facebook, X, and blog posts through user interactions (Salloum and Habash, 2012). Therefore, with the availability of systems that deal with different languages and dialects, a major shift in focus has been witnessed in literature to bring dialects together by enhancing proper machine learning translation systems (Sghaier and Zrigui, 2020).

Arabic is one of the languages known for its diversity in linguistics, which includes various dialects from different countries all over the Arab world. Notably, Dialectal Arabic (DA) consists of different Arabic dialects. It is an informal language that is used in daily life and social media platforms in contrast with Modern Standard Arabic (MSA), also known as “Fushaa,” which is used in formal communications (Harrat et al., 2019). Hence, making the comprehension of different dialects presents a greater challenge compared to MSA, due to its regional variability, especially in the applications of cross-dialect communications, and in sectors such as education and content localization (Sghaier and Zrigui, 2020).

Large language models (LLMs) are a vital approach to understand and enhance the language intelligence of devices (Hadi et al., 2023). LLMs can react to free-text queries without being specifically trained in the activity at hand, which has sparked both excitement and skepticism among researchers regarding their application (Hadi et al., 2023). Models such as OpenAI GPT and Google Bard (Gemini) are examples of LLMs, where they are trained on enormous volumes of text data and can generate human-like prose, answer questions, and perform other language-related tasks with great accuracy (Kasneci et al., 2023). To begin with, OpenAI GPT is a decoder-based, generative pre-trained LLM. It employs an auto-regressive language model that allows sequential text generation. Among many of the advantages present in GPT, one main advantage is that it is a multilingual model, including the Arabic language (Alyafeai et al., 2023). However, it is not an open-access model and is not free of cost. Therefore, developers and researchers have to pay a certain amount based on the number of tokens used per request and the type of model to be used for fine-tuning (Steele, 2023). As for Bard (Gemini), it is developed by Google and is also multilingual; in total, it contains 41 languages (Kadaoui et al., 2023). Similar to GPT, Bard (Gemini) has a certain cost based on the number of tokens used per request and the type of model to be used (Kadaoui et al., 2023). Hence, by analyzing their differences and similarities, a comparison between both models is performed to assist systems in easily translating dialects and achieve human-like reading and writing, building on the comprehensive overview of LLM capabilities by Hadi et al. (2023).

Researchers have been using these models in analyzing various NLP tasks, such as psychological studies of sentiments using GPT (Kheiri and Karimi, 2023). In addition, comparisons with other models such as Bidirectional Encoder Representations from Transformers (BERT) (Zhang et al., 2020) and Bidirectional Long-Form Overlap for Optimizing Multilingual and zero-shot (BLOOMZ) (Yong et al., 2022) have been made in contexts such as translation efficiencies using different languages (Bhat et al., 2023). On the other hand, comparisons between GPT 3.5, GPT 4, and Bard (Gemini) have been made regarding their machine translation (MT) proficiency across 10 varieties of Arabic (Kadaoui et al., 2023). Their analysis shows that LLMs may encounter challenges with dialects for which minimal public datasets exist, but on average, they are better translators of dialects than existing commercial systems. In a similar vein, GPT 4 outperformed Bard (Gemini) in dialect-based commercial systems and different supervised baselines employing zero-shot prompts.

Originally, researchers' main focus was to address the translation of English to Arabic and vice versa (Khoshafah, 2023). However, more recently, researchers have been studying the influence of MSA on the similarity between dialects spoken, as was done by Abu-Haidar (2011) in Baghdad, and vice versa, where researchers study the translation from DA to MSA. For instance, Sghaier and Zrigui (2020) performed a similar study in 2020 where an MT system that translates Tunisian dialect text to MSA using a rule-based approach showed promising results for their proposed solution. Since OpenAI GPT released different models with different versions, researchers have focused on having a comparison between these different versions, where Alyafeai et al. (2023) have compared some of these models, such as GPT 3.5 and GPT 4, on seven distinct Arabic NLP tasks and found that GPT 4 outperforms GPT 3.5 on five NLP tasks. GPT 3.5 and GPT 4 performances were also studied using the Tunisian, Jordanian, and English languages, and the study results highlight a critical dialectical performance gap in GPT, underlining the need to enhance linguistic and cultural diversity in AI models' development, particularly for health-related content (Sallam and Mousa, 2024).

The purpose of this study is to compare the performance of four language models, GPT (versions 3.5, 4, and 5) and Bard (Gemini), in translating a wide corpus of MSA to DA. This novel study bridges a significant gap in understanding model performance across diverse linguistic situations by including a wide corpus of dialects, consisting of over 15 Arabic dialects, in the analysis while evaluating several metrics. Furthermore, two different datasets will be used to further strengthen the analysis using different prompting techniques (zero-shot and few-shot). To explore whether these techniques enhance the quality of dialect translation, zero-shot will be applied to all countries, whereas few-shot will be applied to the weakest country.

This study sheds light on the adaptability and efficiency of these models through careful metric assessments, which is critical for expanding NLP applications in various Arabic-speaking regions. Two datasets are used in this study the first is the Qatar Computing Research Institute (QCRI) Arabic Dialects Identification (QADI) dataset, which contains 18 different countries with their own dialects. QADI contains over 500,000 tweets from social media platforms, spanning 18 different Arabic dialects (Abdelali et al., 2020). Second, the Multi-Arabic Dialect Applications and Resources (MADAR) corpus dataset is used, which includes a large parallel corpus of 25 Arabic city dialects in the travel domain. These are the most popular datasets adapted for studies with Arabic dialects.

This research study aims to answer the following questions:

- How efficient are GPT 3.5, GPT 4, GPT 5, and Bard (Gemini) in translating MSA to different DA in terms of different performance metrics, such as cosine similarity, semantic universal encoder, sentence BERT, similarity encoder, translation error rate (TER), recall-oriented understudy for gisting evaluation (ROUGE), bilingual evaluation understudy (BLEU), and analysis of variance (ANOVA)?
- How consistent is the LLM performance in the MSA translation to different DAs? (e.g., Levantine vs. Gulf vs. Maghrebi)
- How do prompting techniques (zero-shot vs. few-shot) and external factors like sentence length impact the translation accuracy of LLMs?

The main contribution of this study could be summarized as follows:

- It sheds light on the strengths and drawbacks of the GPT 3.5, GPT 4, GPT 5, and Bard (Gemini) models in dealing with DA differences by analyzing their translation quality and accuracy (measured by metrics) and consistency/reliability, across various dialects from MSA. Hence, exploring how LLMs handle dialectal diversity in Arabic.
- It employs various prompt analysis techniques to evaluate the performance of GPT 3.5, GPT 4, GPT 5, and Bard (Gemini), aiming to understand the specific conditions under which each model excels.
- The study's findings fill in a significant gap in research on MSA to dialect translation using LLMs by using a wide corpus of Arabic dialect translations and analyzing GPT 3.5/4/5, and Bard (Gemini) in translating various dialects using different prompting techniques (zero-shot and few-shot).

Therefore, the study relies on it being the first to offer a comprehensive evaluation of LLMs in translating MSA to a wide range of dialects using QADI and MADAR datasets. Moreover, the evaluation of GPT 3.5, GPT 4, GPT 5, and Bard (Gemini) contributes to fine-tuning and developing inclusive NLP tools to serve a larger Arabic-speaking population with diverse dialects. It identifies the strengths and weaknesses of LLMs in different DAs by translation from MSA. Such insights are essential for the development of inclusive NLP tools that can effectively utilize MSA and different DAs in spoken Arabic to enhance digital accessibility and communication. To the best of our knowledge, we are the first study comparing prominent LLMs specially GPT 5 on MT task from MSA to DA over 15 countries.

The remainder of this study is organized as follows: The related work is described in Section 2, and the proposed methodology is detailed in Section 3. Experimental results are reported and analyzed in Section 4. Finally, the concluding remarks and future research directions are described in Section 5.

## 2 Related work

This section highlights the challenges of processing the Arabic language and its dialects in Section 2.1, followed by Section 2.2, which explains and explores different LLMs and Section 2.3 describes various MT approaches.

### 2.1 Challenges for processing Arabic and its dialects

Contemporary Arabic consists of different varieties such as MSA, the official language of the Arab world that is used in formal settings, and dialects of different countries that are commonly used in different informal contexts. In general, Arabic is a complex language with a rich inflectional morphology expressed both templatically and affixationally, as well as various attachable clitic classes (Wright and Caspari, 2011). The dialects of different countries differ from MSA in terms of phonology, morphology, and, to some extent, syntactically, where the differences are based on the presence of clitics and affixes, unlike MSA, are widely used (Salloum and Habash, 2012). Dialects are considered to share all of MSA's problems when it comes to NLP (e.g., optional diacritics and spelling inconsistencies). However, adding to these problems, the absence of standard orthographies for the dialects and their diverse variants, which in turn pose additional issues (Guellil et al., 2021). In addition, there are very few Arabic dialects of English corpora and even fewer dialects of MSA parallel corpora, which makes the number of morphological analyses and tools for these dialects constrained (Salloum and Habash, 2012).

These linguistic challenges pose different difficulties for LLMs in MT. Unlike the English language, which dominates the training of most LLMs, different Arabic dialects are widely underrepresented (Alyafeai et al., 2023; Khondaker et al., 2023). Research papers comparing LLM performance between different languages such as English and Arabic address this gap and confirm it by showing that LLMs achieve better scores in English translation than in Arabic (Peng et al., 2023). Furthermore, within Arabic itself, MSA is better handled in LLMs than in different dialects (Kadaoui et al., 2023). These demonstrate that the wide variation of dialects in the Arabic language and their complexities pose a challenge in MT. Hence, understanding of LLMs ability to translate MSA to different dialects along with the strengths and weaknesses of LLMs in different DAs needs to be addressed as it is critical in the development of NLP tools.

### 2.2 Large language models

LLMs have exhibited a remarkable transformation throughout the years, where they have evolved from generating only natural texts to understanding them through AI (Jiang et al., 2020). LLMs are trained to predict the next token in a sequence based on the context, making the generated outputs coherent. They are able to capture long-range dependencies and perform complex tasks such as translation, summarization, and question answering. Moreover, LLMs can generalize across different domains and diverse dialects through prompting techniques (Alabdullah et al., 2025). Research studies vary in terms of whether to include prompts in the analysis or not. For example, Lilli (2023) has studied ChatGPT 4 using Italian dialects; however, the analysis was done using zero-shot analysis only, and the results showed that the model exhibits a significant gap in analytical skills and struggles with text production and interactive tasks, suggesting superior passive linguistic capabilities compared to active ones. Similarly, GPT 4, GPT 3.5, and Bard (Gemini) were compared in terms of Inductive, Mathematical, and Multi-hop Reasoning Tasks using zero-shot, and GPT 4 was found to be better in all of them compared to GPT 3.5 and Bard (Gemini) (López Espejel et al., 2023). Currently, LLMs are widely used in evaluating the performance of NLP tasks in different languages (Kadaoui et al., 2023). However, LLMs are known to have some issues with rare or unseen words, the problem of overfitting, and the difficulty in capturing complex linguistic phenomena.

Researchers have been evaluating different LLM techniques to shed light on future research in the domain (Chang et al., 2023). Other multilingual models such as XGLM (De Varda and Marelli, 2023) have also been studied and were shown to improve significantly in terms of translation performance. It was found that the model performs best if the answer is estimated based on the probability of the first token in the generated answer. However, these models are yet to be studied further (Zhu et al., 2023). Models such as BERT (Devlin et al., 2018) have also been analyzed in terms of language analysis, such as the Arabic language. However, due to its weakness in Arabic dialects, researchers (Baert et al., 2020) created an enhanced language model (BAERT) that showed better performance than BERT in sentiment analysis. LLM research remains a prominent topic across multiple disciplines, including the development and customization of LLMs tailored to specific languages, dialects, or tasks (Mashaabi et al., 2024). There are various LLMs that support the Arabic language, with GPT being the most prominent. Some researchers suggest that ArabianGPT, specifically designed for Arabic, aligns better with Arabic language and rules (Koubaa et al., 2024).

### 2.3 Machine translation approaches

Machine translation (MT) is an example of an NLP task that addresses grammatical, semantic, and morphological elements between the source and output languages. Importantly, it becomes a challenging task when those elements are significantly different (Joshi et al., 2024). The need for MT systems has been increasing due to the large dialects available on the Internet and their usage in various fields (Sghaier and Zrigui, 2020). Researchers have been studying LLM MT capabilities around the world for different languages. For instance, English to Japanese MT was tested on mBART50, m2m100, Google Translation, Multilingual T5, GPT-3, ChatGPT, and GPT 4 using BLEU, Character Error Rate (CER), WER, Metric for Evaluation of Translation with Explicit ORdering (METEOR), and BERT score, as well as qualitative evaluations by four experts. The analysis showed that GPT 4 outperformed all other models in MT from English to Japanese (Chan and Tang, 2024). Due to their grammatical structure, DA forms a challenge for MT systems (Baniata et al., 2022). MT is an example of an NLP task that addresses grammatical, semantic, and morphological elements between the source and output languages. Importantly, it becomes a challenging task when those elements are significantly different (Joshi et al., 2024). Several approaches and tools are available to perform MT, such as rule-based approaches, hybrid approaches, and sequence-to-sequence (seq2seq) models as well as LLMs (Okpor, 2014). For instance, Salloum and Habash (2012) created a rule-based approach system to translate DA to MSA, which depends on a morphological analyzer, transfer rules, and dictionaries to generate sentences and choose the best matches.

Several researchers have widely used the rule-based approach to translate Arabic dialects to MSA (Al-Gaphari and Al-Yadoumi, 2010; Hamada and Marzouk, 2018; Bouamor et al., 2014). Another study created a hybrid approach to translate the Egyptian dialect to MSA and achieved 90% performance through tokenization (Bakr et al., 2008). Beyond these, Hamed et al. (2025) developed Lahjawi, a customized model specialized in cross-dialectal translation (DA to MSA) that supports 15 dialects. Lahjawi was trained on 7 well-known datasets, including MADAR and Parallel Arabic Dialectal Corpus (PADIC), and fine-tuned above a small language model - Kuwain 1.5B. The model achieved adequate BLEU scores and an accuracy of 58% based on human evaluation. Moreover, Alimi et al. (2024) developed MT model to translate DA to MSA. The model was trained on MADAR and PADIC datasets and fine-tuning transformers such as T5X and AraT5 and some existing tools. The best translation results revealed were for Levantine and Maghrebi region dialects. Some authors also adapted a hybrid approach to translate the Moroccan dialect to MSA using processing tools for MSA (Ridouane and Bouzoubaa, 2014; Hamada and Marzouk, 2018), whereas other studies focused on Neural Machine Translation (NMT) for Arabic dialects (Baniata et al., 2018; Guellil et al., 2017). For example, Baniata et al. (2022) developed an NMT model to translate DA to MSA through multi-head attention with reverse positional encoding and sub-word units. The model achieved high BLEU scores, proving their encoding method across several datasets. In addition, other researchers expand the Dial2MSA dataset through seq2seq datasets in different domains, including social media covering different regions. Leaving a reliable NMT training, the authors conducted a performance evaluation, and it was found that AraT5 achieved the highest performance (Khered et al., 2025). Moreover, researchers Alabdullah et al. (2025) evaluated six LLMs on DA to MSA translation, including Levantine, Egyptian, and Gulf Dialects using different prompting techniques. They demonstrated that GPT 4o achieved the highest score in translation performance, while a fine-tuned version of Gemma2-9B achieved a higher CHrF++ score compared to GPT 4o in zero-show prompting.

Furthermore, researchers utilized LLMs to perform MT tasks. For instance, Zhu et al. (2023) evaluated the multilingual translation of four LLMs, namely, GPT, XGLM, OPT, and BLOOMZ. Interestingly, the researchers found that such models adapt new patterns to translate. GPT proved excellent capability in MT and outperformed Google Translate according to Peng et al. (2023). In addition, the AraFinNLP shared tasks highlight critical challenges and discussions for cross-dialect translation in preservation of intents using the known ArbBanking77 dataset. The findings highlight that accurate MSA to DA (Moroccan, Tunisian, and Palestinian) translation is possible yet challenging. They demonstrated that fine-tuned BERT models and data augmentation achieve high performance in handling Arabic dialects for financial applications (Malaysha et al., 2024). Moreover, SHAMI-MT developed bidirectional MT models built on the AraT5v2 model and fine-tuned on the Nbra corpus. They evaluated the translation between MSA and the Syrian dialect and used MADAR for benchmark (Sibaee et al., 2025). Similarly, Mohamed et al. (2012) presented a method to convert MSA to Egyptian dialect, applied on part-of-speech (POS). They showed that such MT task improves tagging and is considered as valuable training data for underrepresented dialects.

Prior research studies addressed the translation from MSA to different dialects. A study conducted empirical analysis focusing on Arabic-based LLMs to assess their ability to translate DA to MSA, utilizing four datasets with English-based LLMs as a baseline (Jibrin et al., 2025). They highlighted that AceGPT and Jais performed the best BLEU scores across all data sets, establishing their reliability in Arabic formality. In another study, GPT was evaluated on various NLP tasks. It was revealed that GPT, in comparison with BLOOMZ, struggles on some Arabic tasks yet comparable to human judgment (Khondaker et al., 2023). Several studies explored this field with more precision in relation to the Nuance Arabic Dialect Identification (NADI) 2023 competition. Demidova et al. (2024) performed sentence-based translation from DA to MSA across four dialects through Jais, No Language Left Behind (NLLB), GPT 3.5, and GPT 4 LLMs. They found that Jais outperforms the other models consistently, achieving high BLEU scores whereas NLLB was the least performer. Similarly, other researchers mainly focused on fine-tuning LLama-3 with 8B parameters through Parameter Efficient Fine-Tuning (PEFT) and Low Rank Adaptation (LoRA) methods. The task was also DA-MSA translation across four datasets. LLama fine-tuned model exhibits strong performance related to BLEU metric. Moreover, the 6th Workshop on Open-Source Arabic Corpora and Processing Tools (OSACT) showed interesting findings through different studies specifically for Dialect to MSA MT task including 5 dialects. (Atwany et al. 2024) evaluated AraT5, NLLB, and GPT 3.5. The results show that fine-tuning Arat5 and NLLB on the MADAR dataset demonstrates low BLEU scores, whereas prompting GPT 3.5 achieved high BLEU scores. Moreover, other researchers used GPT 3.5 for dataset generation (Abdelaziz et al., 2024). They used the Saudi Audio Dataset for Arabic (SADA) to translate the audio dialects to MSA texts, leading to notable performance in machine translation achieving high BLEU scores between 25.5 and 31.5. Alahmari et al. (2024) fine-tuned four versions of AraT5 model highlighting that AraT5v2-base-1024 model achieved the highest BLEU score of 21.0. Various researchers have utilized MT with a special focus on the context of Arabic dialects. Table 1 summarizes the MT approaches proposed by the researchers.

**Table 1:** Summary of machine translation (MT) approaches for Arabic dialects.

**Research**	**Dialect(s)**	**Approach**
Bakr et al., 2008	Egyptian → MSA	Hybrid
Al-Gaphari and Al-Yadoumi, 2010	Sana'ani → MSA	Rule-based
Salloum and Habash, 2012	Arabic Dialects → MSA	Rule-based
Mohamed et al., 2012	MSA → Egyptian	Rule-based
Bouamor et al., 2014	Mainly Egyptian	Rule-based, Corpus of 2,000 sentences
Ridouane and Bouzoubaa, 2014	Moroccan → MSA	Hybrid
Guellil et al., 2017	Algerian	NMT
Hamada and Marzouk, 2018	Egyptian → MSA	Hybrid/Rule-based
Baniata et al., 2018	Arabic dialects → MSA	Neural MT (NMT)
Hamed et al., 2025	15 Dialects → MSA	Custom cross-dialectal model
Alimi et al., 2024	Levantine, Maghrebi → MSA	Transformer-based MT (AraT5, T5X)
Alabdullah et al., 2025	Levantine, Egyptian, Gulf → MSA	LLM-based MT (GPT 4o, Gemma2-9B)
Zhu et al., 2023	Multilingual/Arabic	LLM-based MT (GPT, XGLM, OPT, BLOOMZ)
Malaysha et al., 2024	Moroccan, Tunisian, Palestinian → MSA	LLM + fine-tuned BERT
Sibaee et al., 2025	Syrian → MSA	AraT5v2-based bidirectional MT
Khered et al., 2025	Arabic Dialects → MSA	Seq2seq / Transformer (AraT5)
Jibrin et al., 2025	Arabic Dialects → MSA	LLM-based MT (AceGPT, Jais)
Khondaker et al., 2023	Arabic Dialects → MSA	LLM-based MT (GPT, BLOOMZ)
Demidova et al., 2024	Egyptian, Emirati, Jordanian, and Palestinian → MSA	LLM-based MT (Jais, NLLB, GPT 3.5, GPT 4)
Atwany et al., 2024	Gulf, Egyptian, Levantine, Iraqi and Maghrebi → MSA	LLM-based MT (AraT5, NLLB, GPT 3.5)
Abdelaziz et al., 2024	Saudi Dialect → MSA	LLM-based MT (GPT 3.5)
Alahmari et al., 2024	Arabic dialects → MSA	Transformer MT (AraT5v2)

## 3 Proposed methodology

This section discusses the chosen dataset in Section 3.1, followed by Section 3.2, which describes the prompting techniques. Model selection is mentioned in Section 3.3, and the chosen performance metrics are detailed in Section 3.4.

### 3.1 Dataset

Translating Arabic dialects has been a wide area of research (Harrat et al., 2019). In our research, we aim to use the QADI dataset and the MADAR corpus dataset. QADI dataset is a pre-processed dataset collected through X media platform, and it includes 18 dialects from different Arab countries, the dataset is already cleaned and has no hashtags, emojis, or such symbols which might affect the translation quality (Abdelali et al., 2020). The dataset has 540k training tweets and 3,303 test tweets in total. The rationale for choosing the QADI dataset is the large number of dialects it has which will help us address our research questions and compare the performance evaluation of LLMs. However, in the current study, 50K samples will be used from all countries for the analysis due to computational resource restrictions. We applied random sampling, the QADI dataset was balanced across dialects, our random selection ensured that the selected 50K tweets have no bias and ensure equal selection among the sentences. Table 2 shows different country codes using ISO-3166-1 with corresponding users and tweet count of QADI dataset.

**Table 2:** QADI dataset: users and tweet counts by country using ISO-3166-1 codes.

**Country**	**Users**	**Training tweets (k)**	**Test tweets**
Iraq (IQ)	142	18.4	178
Bahrain (BH)	169	28.3	184
Kuwait (KW)	160	49.9	190
Saudi Arabia (SA)	149	35.4	199
United Arab Emirates (AE)	172	27.8	192
Oman (OM)	176	24.8	169
Qatar (QA)	139	36.7	198
Yemen (YE)	138	11.6	193
Syria (SY)	139	18.3	194
Jordan (JO)	146	34.1	180
Palestine (PL)	145	48.6	173
Lebanon (LB)	141	38.4	194
Egypt (EG)	150	67.8	200
Sudan (SD)	139	16.3	188
Libya (LY)	149	40.9	169
Tunisia (TN)	68	12.9	154
Algeria (DZ)	130	17.6	170
Morocco (MA)	73	12.8	178

Similarly, the MADAR corpus dataset (Bouamor et al., 2019) contains 25 cities representing 15 countries, each with a unique dialect where some countries feature multiple cities (e.g., Egypt has Aswan, Cairo, and Alexandria) with 2K samples from each dialect. The advantage of using the MADAR dataset is that it includes MSA baseline translation for the sentences present inside the dialects of each country. Hence, making the evaluation of GPT and Bard (Gemini) stronger by comparing the results of these models with the baseline given within the dataset. This study will analyze 15 countries from the MADAR dataset primarily focusing on the capitals of countries that are also included in QADI. Table 3 shows all the city dialects from the MADAR dataset, showing the different cities with their dialects from various Arabic countries.

**Table 3:** All the city dialects and regions that were included in the building of the MADAR dataset.

**Region**	**Sub-region**	**Cities**	**Codes**
Maghreb	Morocco	Rabat, Fes	RAB, FES
	Algeria	Algiers	ALG
	Tunisia	Tunis, Sfax	TUN, SFX
	Libya	Tripoli, Benghazi	TRI, BEN
Nile Basin	Egypt	Cairo, Alexandria, Aswan	CAI, ALX, ASW
	Sudan	Khartoum	KHA
Levant	South Levant	Jerusalem, Amman, Salt	JER, AMM, SAL
	North Levant	Beirut, Damascus, Aleppo	BEI, DAM, ALE
Gulf	Iraq	Mosul, Baghdad, Basra	MOS, BAG, BAS
	Gulf	Doha, Muscat, Riyadh, Jeddah	DOH, MUS, RIY, JED
Yemen	Yemen	Sana'a	SAN

### 3.2 Prompting techniques

**Figure 1:**
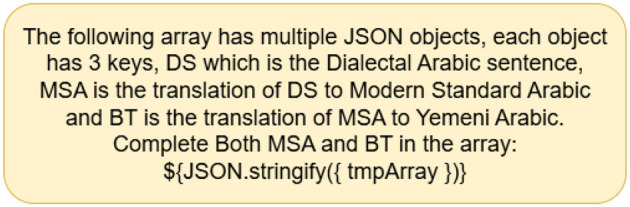
Zero-shot prompt - QADI.

**Figure 2:**
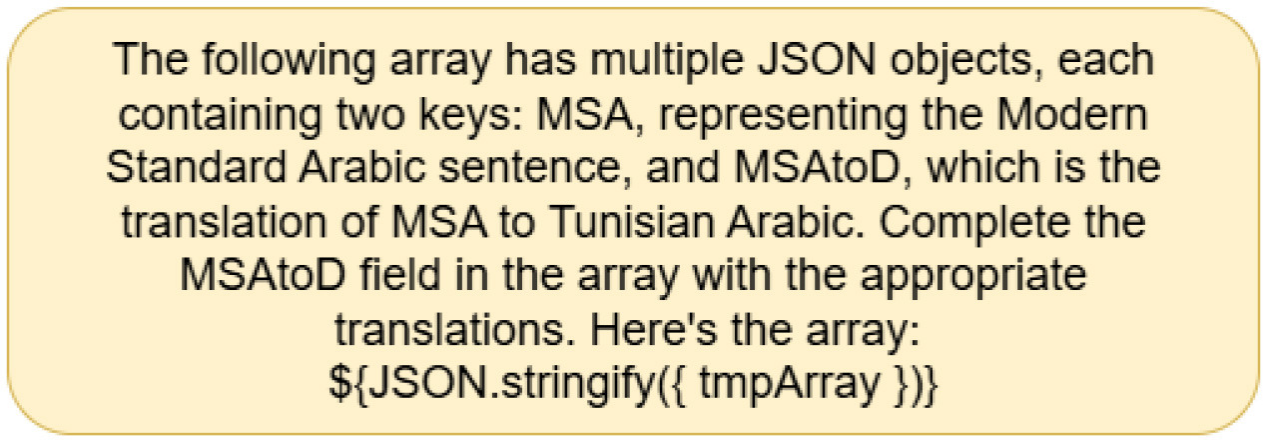
Zero-shot prompt - MADAR.

**Figure 3:**
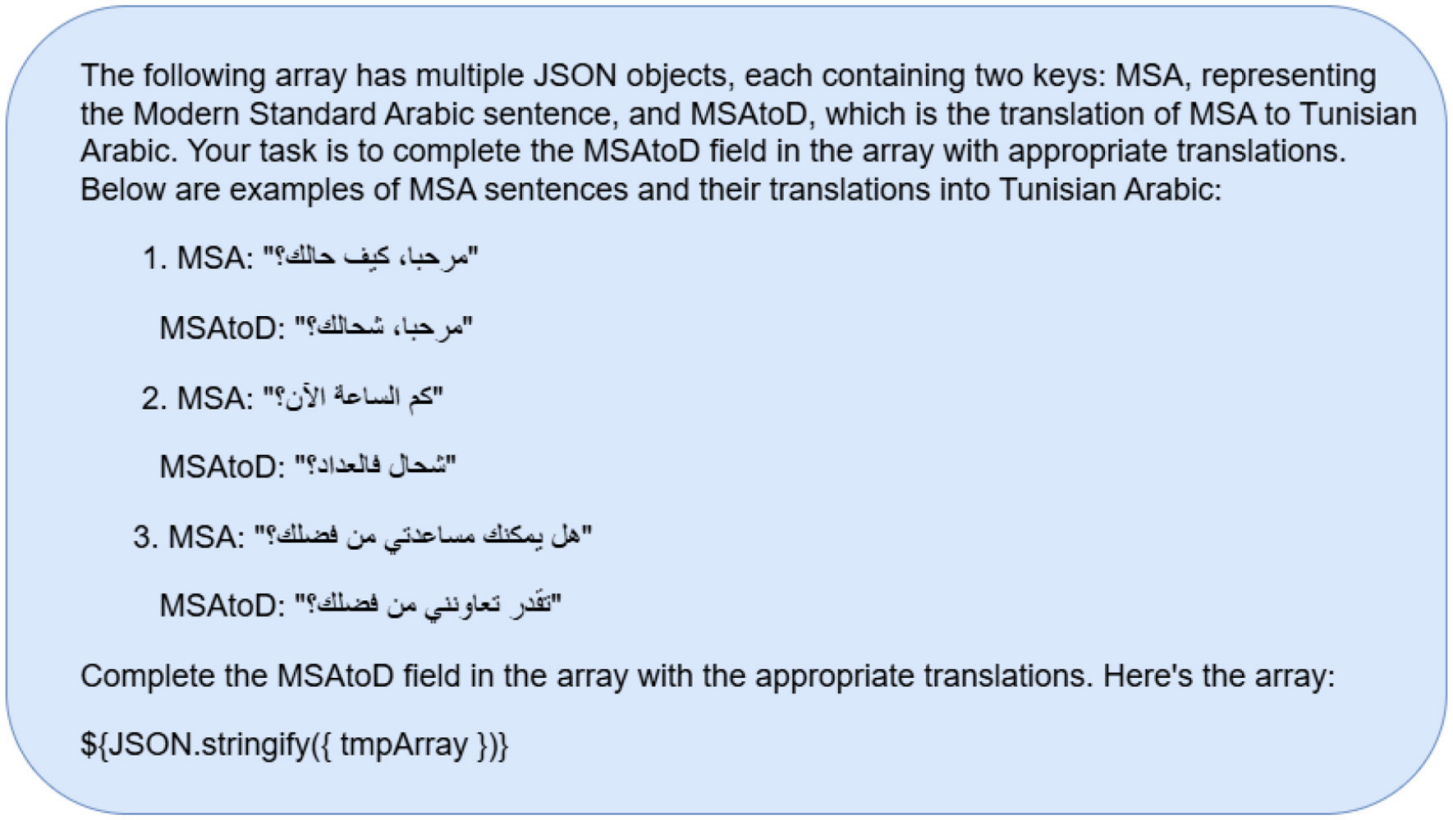
Few-shot prompt - MADAR.

Prompting strategies have been developed to optimize LLMs' performance and outcomes. The most frequent of these tactics are zero-shot and few-shot. The zero-shot prompt plainly describes the task and provides information without examples (Allingham et al., 2023). Figures 1, 2 show an example of the prompts used to perform the translation task. Unlike zero-shot prompts, few-shot prompts include data examples and sample responses (Jiang et al., 2022). On the other hand, a few-shot prompting technique is established by providing an example within the prompt itself, where one-shot includes a single example, two-shot includes 2 examples, etc. We will include both zero-shot and few-shot prompts. As well as a few shot prompts (one-shot) for the country with the weakest dialect translation given by the models to check whether including an example within the prompt would enhance the overall accuracy of the translation. An example of a prompt is shown in Figure 3 to test whether the models would provide a better translation as compared to zero-shot approaches.

### 3.3 Model selection

This research paper will be using OpenAI's most recent model GPT 5 along with GPT 3.5, GPT 4, and Google's Bard (Gemini) “text-bison” model due to their exceptional performance in research (Zhu et al., 2023; Peng et al., 2023; Khondaker et al., 2023; Kadaoui et al., 2023). LLMs are widely used to evaluate the performance of Arabic NLP tasks such as GPT 3.5, GPT 4, Bard (Gemini), XGLM, and OPT (Zhu et al., 2023). To save computational cost and time, GPT 5 will only be ran on MADAR dataset, whereas QADI will include all remaining models. This study's selection criteria for the models aim to balance between budget and computing resources. In addition, LLM languages that do not include the Arabic language, such as Falcon-7b (Penedo et al., 2023), were initially excluded from the search scope of suitable LLMs. A brief summarization of both models is shown in Table 4.

**Table 4:** Tabular comparison between GPT and Bard.

**Aspect**	**GPT 3.5**	**GPT 4**	**GPT 5**	**Bard**
Source	OpenAI	OpenAI	OpenAI	Google
Language model	GPT 3.5- turbo- 16k	'GPT 4-0125-preview'	'GPT 5'	'text-bison'
Model architecture	Transformer decoder based	Transformer decoder based	Transformer decoder based	Transformer based
Availability	Limited free access	Paid	Paid	Limited free access
Languages	Multilingual	Multilingual	Multilingual	Multilingual
Parameter Size	175 Billion	1.76 Trillion	Not Announced	137 Billion

**Figure 4:**
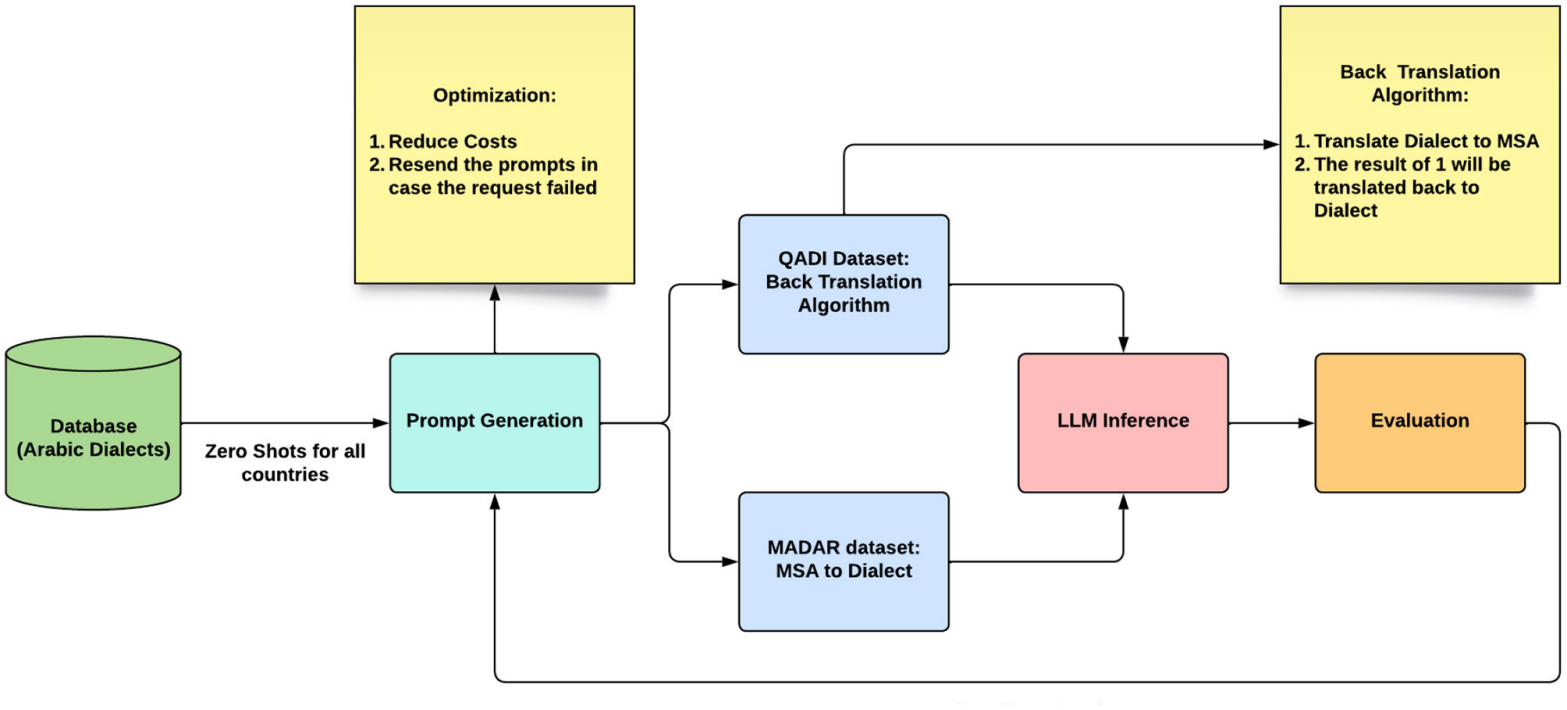
Experiment pipeline.

Figure 4 shows the experiment pipeline implemented for GPT and Bard (Gemini). The experiment starts using the data in the dataset as a prompt for each LLM. Initially, all prompts will be applied with zero-shot techniques, meaning that no example will be included within the prompt. However, after performing the analysis, the country with the least translation performance will be analyzed again but with the few-shot prompting technique. In the QADI dataset, to have a baseline to compare the LLM results with, the back translation process is used (Behr, 2017), where dialects are translated to MSA; then, the resulting MSA is translated back to the corresponding dialect to compare the final resulting dialect with the original dialect from the dataset. However, MADAR offers a baseline for dialects and MSA; therefore, no back-translation will be needed.

For LLM inference, we used the code provided on the Application Programming Interface (API) websites with some correction techniques; rerunning the prompt if the model returns an error to ensure a correct response. After doing so, the error rate in the resulting samples has dropped sufficiently. Cost optimization technique has also been adapted by running 10 translations per API request, which reduced the cost. A threshold of 10 requests was set as the maximum accumulation; as the threshold increases, the error rate also increases. Finally, the experiment results will be evaluated by calculating the selected performance metrics described in the upcoming section.

### 3.4 Performance metrics

We aim to quantify the differences in performance between GPT 3.5, GPT 4, GPT 5, and Bard (Gemini) and to determine how these models can perform the translation task given the complexity of the Arabic language. There are various common evaluation metrics for comparison. The present study will use 7 evaluation metrics (i.e., cosine similarity, sentence BERT, semantic universal encoder, TER, BLEU, ROUGE, and ANOVA test). These metrics were chosen based on their strengths and popularity in analyzing Arabic sentences. To attest for normality, the Shapiro–Wilk test was used for ANOVA (Alabdullah et al., 2025).

One of the common MT metrics is the universal similarity encoder, which is a neural network architecture for learning similarity-preserving embeddings that uses pre-trained embeddings (e.g., Word2Vec, GloVe, or BERT embeddings) to compare two sentences, rather than having a specific calculation formula. Its range varies from –1 to 1, where results closer to 1 are indicative of high semantic similarity.

However, cosine similarity calculates the cosine of the angle formed by two vectors that represent phrases in several dimensions that represent a word or contextual information. Equation 1 below shows the cosine similarity, where A and B are vectors.


(1) $$\begin{array}{l} \text{Cosine similarity}=\frac{A\cdot B}{\left||A||\cdot ||B|\right|} \end{array}$$

High positive values in cosine similarity (close to 1) indicate that there is great similarity between the two vectors.

Sentence BERT is a transformer that adapts cosine similarity by using Tensorflow. The general process involves encoding sentences into fixed-size vectors using pre-trained BERT embedding and then calculating a similarity score between these vectors (Mrinalini et al., 2022). Since sentence BERT adapts cosine similarity, it follows the same metric measures of –1 to 1, where close values to –1 mean that the two vectors are completely dissimilar, and values close to 1 mean that there is a high similarity between the vectors. The universal sentence encoder finds the similarity between sentences based on semantics, where it is used to convert phrases into dense vector representations.

Finally, the TER metric is specifically used for MT tasks by comparing the MT outputs against human-generated translation to assess the quality of MT outputs, as shown in Equation 2.


(2) $$\begin{array}{l} \text{TER}=\frac{\text{Total edits}}{\text{Total words in reference translation}} \end{array}$$

A lower TER score indicates a better translation quality as it means that fewer edits are needed to align the machine-generated translation.

Moreover, the BLEU metric is a widely popular metric used in research (Sallam and Mousa, 2024) where individual translated segments, usually sentences, are scored by comparing them with a collection of high-quality reference translations. These scores are then averaged throughout the entire corpus to provide an approximation of the translation's overall quality (Papineni et al., 2002). It aims to find the similarity between the translated text and the reference sentence by employing n-grams; contiguous group of n-words that are similar. The metric values range from 0 to 1, and typically a higher value means that more words are overlapping between the machine-translated sentence and the referenced sentence, as shown in Equation 3 (Papineni et al., 2002).


(3) $$\begin{array}{l} {\text{BLEU}}_{w}\left(Ŝ;S\right):=\text{BP}\left(Ŝ;S\right)\cdot \exp\left(\overset{\infty }{\underset{n=1}{\sum }}w_{n}\log p_{n}\left(Ŝ;S\right)\right) \end{array}$$

where BP is the brevity penalty, w is the weights for each n-gram, and p is the precision of n-grams.

Furthermore, ROUGE is a collection of metrics and software packages for assessing automatic summarization and MT software in natural language processing. The metrics assess an automatically generated summary or translation to a reference or a collection of references (human-created summary or translation). ROUGE measures range from 0 to 1, with higher scores indicating a stronger resemblance between the automatically generated summary and the reference (Lin and Hovy, 2003).

ANOVA is a statistical approach for comparing the means of three or more samples to determine whether one of them is substantially different from the others (Keselman et al., 1998). It accomplishes this by analyzing the variance in the data and categorizing it as the variance between groups and the variance within groups. The *p*-value is calculated using the ANOVA test statistic, also known as the F-statistic, as shown in Equation 4.


(4) $$\begin{array}{l} \text{F-statistic (ANOVA Coefficient)}= \end{array}$$

$$\begin{array}{l} \frac{\text{Mean Sum of Squares due to Treatment (MST)}}{\text{Mean Sum of Squares due to Error (MSE)}} \end{array}$$

The *p*-value indicates whether the differences in group means are statistically significant (Keselman et al., 1998). In this study, since we are performing various analyses and tests, it became important to employ ANOVA to determine the statistical significance of the results.

## 4 Experimental results

This section discusses the model responsiveness in Section 4.1, followed by the metric performance and dialect variations in Section 4.2. Finally, Section 4.3 discusses the impact of sentence length on the model accuracy.

### 4.1 Model responsiveness

In general, in terms of responsiveness, the models were responsive when given a prompt with input. However, there were differences in the output details of both models. GPT gave a direct response where Gemini explained each word in a row.

When running APIs, Bard (Gemini) has shown varying error rates when translating ranging from 5% up to 71%. This error rate was varying based on the load on the network at the execution time and length of the dataset being analyzed. Hence, to reduce the error rate, we ran Bard (Gemini) when the network was not preoccupied with many other tasks and ran the dataset in smaller batches to reduce the chances of error. There were several cases where Bard (Gemini) has either returned the same input as output, empty output, or a message that says that it is unable to handle a given task.

The rate of failing to give an output is most noticeable when performing the back translation from MSA to a certain dialect in QADI dataset. For example, for the back translation for IQ dialect, Bard (Gemini) failed to give an output with the rate of 37.5%, whereas GPT 3.5 has only failed to do so with a 5.6% rate, and GPT 4 had 0.2% error rate. Therefore, a correction technique was added in the code, where the response was checked, if it included an error, resend the same prompt. After doing so, the error rate in the resulting samples has dropped considerably.

### 4.2 Performance metrics and dialect variations

#### 4.2.1 Similarity metrics

This section discusses the similarity metrics and the performance of the LLMs on the MADAR and QADI datasets in terms of universal similarity encoder, cosine similarity, sentence BERT, BLEU, and ROUGE F1 scores. The metrics aimed to assess the efficiency and accuracy of the translation process of different dialects. The analysis explained below is further demonstrated in Tables 5 – 11. To address the research questions, both GPT 3.5/4 and Bard (Gemini) exhibited similar performance levels across the metrics among dialects in both datasets.

**Table 5:** Bard metric similarities mean among 18 dialects from QADI dataset.

**Dialect**	**Univ. Sim. Enc**.	**Cosine Sim**.	**Sent. BERT**	**BLEU**	**ROUGE-L**
JO	0.68	0.43	0.92	0.07	0.43
AE	0.65	0.38	0.92	0.35	0.38
LB	0.67	0.40	0.87	0.38	0.40
IQ	0.64	0.40	0.91	0.39	0.41
BH	0.67	0.46	0.88	0.07	0.46
DZ	0.64	0.41	0.89	0.39	0.41
EG	0.72	0.47	0.89	0.45	0.47
KW	0.67	0.46	0.94	0.43	0.45
LY	0.70	0.48	0.90	0.45	0.47
MA	0.63	0.38	0.94	0.04	0.38
OM	0.64	0.45	0.94	0.43	0.45
PL	0.64	0.42	0.94	0.40	0.42
QA	0.67	0.42	0.94	0.05	0.42
SA	0.65	0.39	0.93	0.37	0.39
SD	0.68	0.44	0.90	0.06	0.43
SY	0.66	0.46	0.90	0.43	0.45
TN	0.65	0.42	0.89	0.39	0.41
YE	0.68	0.47	0.93	0.44	0.47

**Table 6:** Bard metric similarities mean among 15 dialects from MADAR dataset.

**Dialect**	**Univ. Sim. Enc**.	**Cosine Sim**.	**Sent. BERT**	**BLEU**	**ROUGE-L**
JO	0.56	0.34	0.93	0.37	0.32
LB	0.53	0.35	0.93	0.34	0.28
IQ	0.50	0.33	0.93	0.32	0.26
DZ	0.52	0.31	0.93	0.29	0.23
EG	0.57	0.38	0.93	0.37	0.32
LY	0.53	0.32	0.93	0.31	0.25
MA	0.50	0.31	0.93	0.29	0.23
OM	0.58	0.40	0.93	0.38	0.33
PL	0.56	0.39	0.92	0.37	0.32
QA	0.53	0.36	0.93	0.34	0.28
SA	0.53	0.35	0.93	0.33	0.27
SD	0.56	0.38	0.94	0.37	0.32
SY	0.55	0.39	0.93	0.37	0.32
TN	0.48	0.26	0.93	0.25	0.17
YE	0.50	0.28	0.93	0.27	0.20

**Table 7:** GPT 3.5 metric similarities mean among 18 dialects from QADI dataset.

**Dialect**	**Univ. Sim. Enc**.	**Cosine Sim**.	**Sent. BERT**	**BLEU**	**ROUGE-L**
JO	0.66	0.38	0.89	0.43	0.46
AE	0.66	0.37	0.88	0.39	0.43
LB	0.65	0.40	0.94	0.48	0.50
IQ	0.62	0.33	0.84	0.38	0.40
BH	0.67	0.40	0.87	0.44	0.47
DZ	0.59	0.29	0.91	0.28	0.31
EG	0.65	0.35	0.86	0.32	0.35
KW	0.65	0.39	0.90	0.45	0.48
LY	0.63	0.34	0.85	0.32	0.36
MA	0.64	0.34	0.89	0.37	0.40
OM	0.64	0.39	0.84	0.46	0.49
PL	0.67	0.43	0.84	0.53	0.55
QA	0.63	0.35	0.87	0.25	0.40
SA	0.63	0.33	0.89	0.32	0.36
SD	0.65	0.37	0.85	0.35	0.46
SY	0.65	0.39	0.90	0.43	0.46
TN	0.66	0.41	0.83	0.46	0.49
YE	0.63	0.39	0.85	0.43	0.45

**Table 8:** GPT 3.5 metric similarities mean among 15 dialects from MADAR dataset.

**Dialect**	**Univ. Sim. Enc**.	**Cosine Sim**.	**Sent. BERT**	**BLEU**	**ROUGE-L**
JO	0.55	0.35	0.92	0.34	0.30
LB	0.52	0.32	0.91	0.32	0.25
IQ	0.51	0.29	0.93	0.28	0.22
DZ	0.50	0.28	0.93	0.26	0.20
EG	0.54	0.34	0.93	0.33	0.28
LY	0.51	0.27	0.93	0.27	0.20
MA	0.50	0.27	0.93	0.26	0.20
OM	0.53	0.31	0.92	0.29	0.24
PL	0.54	0.34	0.92	0.33	0.28
QA	0.53	0.31	0.93	0.30	0.24
SA	0.55	0.34	0.93	0.34	0.28
SD	0.53	0.31	0.92	0.29	0.24
SY	0.55	0.36	0.92	0.35	0.30
TN	0.48	0.24	0.93	0.23	0.16
YE	0.50	0.26	0.93	0.25	0.19

**Table 9:** GPT 4 metric similarities mean among 18 dialects from QADI dataset.

**Dialect**	**Univ. Sim. Enc**.	**Cosine Sim**.	**Sent. BERT**	**BLEU**	**ROUGE-L**
JO	0.73	0.50	0.82	0.49	0.51
AE	0.71	0.45	0.91	0.44	0.46
LB	0.74	0.50	0.94	0.49	0.51
IQ	0.70	0.43	0.88	0.43	0.45
BH	0.72	0.48	0.91	0.48	0.49
DZ	0.75	0.53	0.91	0.55	0.57
EG	0.77	0.55	0.90	0.55	0.57
KW	0.68	0.45	0.88	0.45	0.47
LY	0.70	0.43	0.87	0.42	0.44
MA	0.70	0.41	0.89	0.40	0.41
OM	0.65	0.39	0.77	0.38	0.39
PL	0.71	0.49	0.88	0.48	0.50
QA	0.66	0.37	0.87	0.36	0.37
SA	0.69	0.38	0.89	0.36	0.38
SD	0.74	0.50	0.93	0.51	0.53
SY	0.72	0.48	0.92	0.46	0.49
TN	0.71	0.44	0.88	0.44	0.45
YE	0.69	0.43	0.91	0.41	0.43

**Table 10:** GPT 4 metric similarities mean among 15 dialects from MADAR dataset.

**Dialect**	**Univ. Sim. Enc**.	**Cosine Sim**.	**Sent. BERT**	**BLEU**	**ROUGE-L**
JO	0.60	0.42	0.93	0.41	0.37
LB	0.54	0.34	0.43	0.36	0.28
IQ	0.54	0.34	0.93	0.33	0.27
DZ	0.51	0.30	0.93	0.29	0.23
EG	0.56	0.38	0.93	0.38	0.33
LY	0.52	0.31	0.93	0.30	0.24
MA	0.47	0.26	0.93	0.25	0.18
OM	0.53	0.33	0.93	0.32	0.26
PL	0.59	0.41	0.92	0.41	0.36
QA	0.57	0.39	0.93	0.38	0.33
SA	0.58	0.41	0.93	0.40	0.35
SD	0.54	0.33	0.93	0.32	0.26
SY	0.59	0.41	0.92	0.41	0.36
TN	0.48	0.26	0.93	0.25	0.18
YE	0.52	0.30	0.92	0.29	0.22

**Table 11:** GPT 5 metric similarities mean among 15 dialects from MADAR dataset.

**Dialect**	**Univ. Sim. Enc**.	**Cosine Sim**.	**Sent. BERT**	**BLEU**	**ROUGE-L**
JO	0.62	0.46	0.93	0.47	0.43
LB	0.58	0.39	0.92	0.39	0.34
IQ	0.55	0.37	0.92	0.37	0.31
DZ	0.50	0.28	0.93	0.26	0.20
EG	0.59	0.44	0.92	0.44	0.40
LY	0.54	0.37	0.92	0.36	0.30
MA	0.56	0.40	0.92	0.39	0.34
OM	0.52	0.34	0.93	0.37	0.28
PL	0.61	0.46	0.92	0.47	0.42
QA	0.59	0.43	0.92	0.44	0.38
SA	0.58	0.42	0.92	0.43	0.38
SD	0.54	0.38	0.92	0.37	0.32
SY	0.62	0.47	0.92	0.49	0.44
TN	0.53	0.34	0.92	0.33	0.27
YE	0.55	0.35	0.93	0.34	0.28

**Figure 5:**
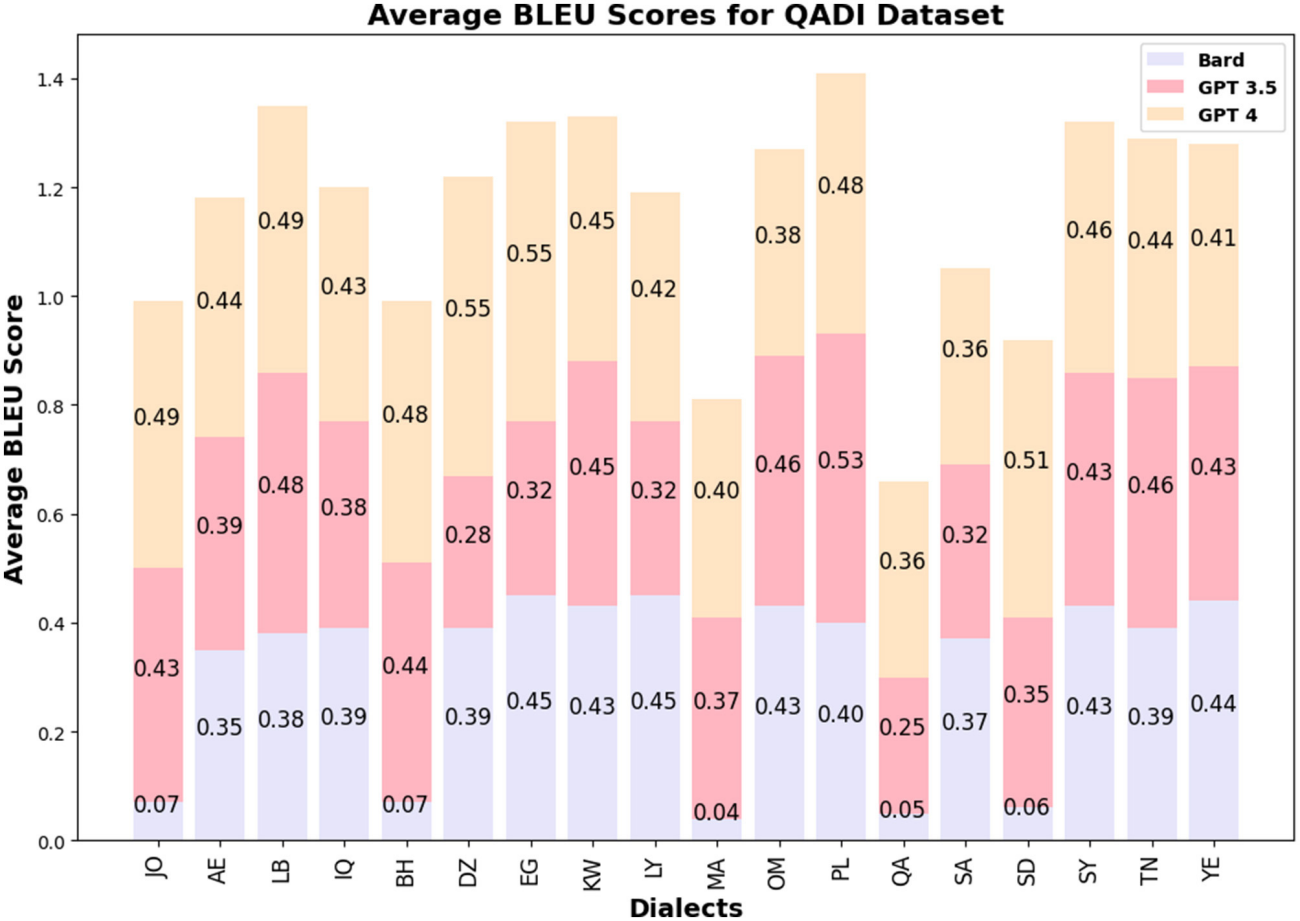
Average BLEU scores QADI.

**Figure 6:**
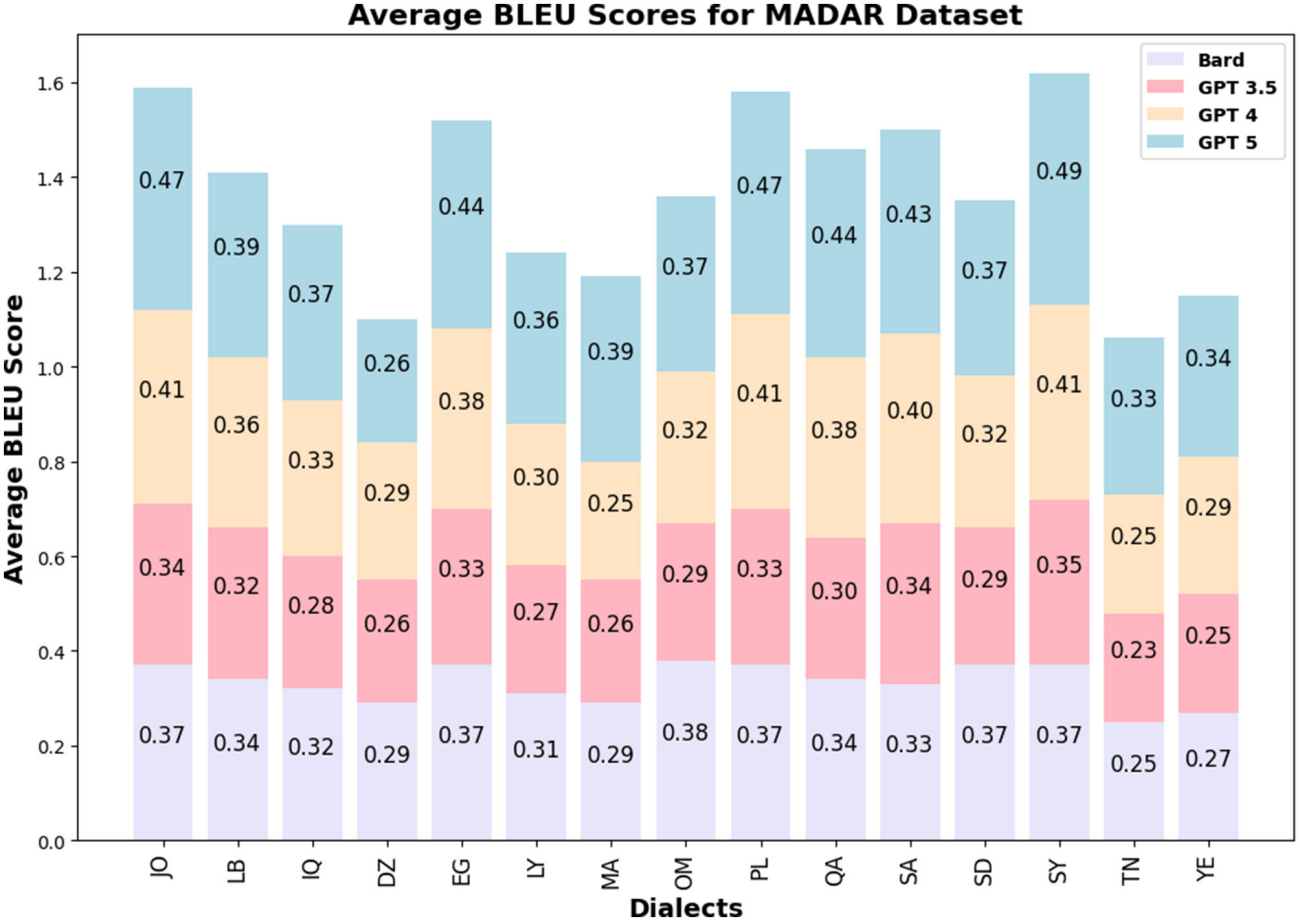
Average BLEU scores MADAR.

The BLEU score values for GPT 3.5/4 are similar among the LLMs and countries for QADI, whereas GPT 5 slightly outperformed its prior models in MADAR dataset. Figures 5, 6 visualize the BLEU scores labeled by each country where the LLMs showed consistent results in MADAR. Bard (Gemini) in the QADI dataset achieved a low score for some countries. These numbers explain that a few words were overlapping between the input and the translated dialect.

Furthermore, when employing a universal similarity encoder and cosine similarity in QADI as shown in Table 12, GPT 4 outperforms the models, which makes it the dominant, followed by Bard (Gemini) and then GPT 3.5. The mean universal similarity encoder score is 71% for GPT 4, 64% for GPT 3.5, and 66% for Bard (Gemini) among all countries. For the MADAR dataset in Table 13, GPT 5 outperforms all models by having a 57% average, whereas GPT 4 has a mean of 54%, GPT 3.5 mean is 52%, whereas Bard (Gemini) has a mean of 53%. This suggests that Bard (Gemini) has shown comparable skill to older GPT models in understanding and conveying the semantic connections among the translated sentences in the MADAR dataset, whereas GPT 5 stands out overall. Whereas for the QADI dataset, GPT 4 had a higher mean, which indicates that it has the best skill in conveying the semantic connections with the existence of the back translation algorithm.

**Table 12:** Average similarity metrics for QADI dataset.

**Metric**	**GPT 3.5**	**GPT 4**	**Bard (Gemini)**
Universal similarity encoder	0.64	0.71	0.66
Cosine similarity	0.37	0.46	0.43
Sentence BERT	0.87	0.89	0.91
BLEU	0.39	0.45	0.31
ROUGE-L	0.43	0.47	0.43
TER	15.62%	15.75%	16.55%

Lower error rates are denoted by green.

**Table 13:** Average similarity metrics for MADAR dataset.

**Metric**	**GPT 3.5**	**GPT 4**	**GPT 5**	**Bard (Gemini)**
Universal similarity encoder	0.52	0.54	0.57	0.53
Cosine similarity	0.31	0.35	0.39	0.34
Sentence BERT	0.93	0.90	0.92	0.93
BLEU	0.30	0.34	0.39	0.33
ROUGE-L	0.24	0.28	0.34	0.27
TER	6.76%	6.74%	6.61%	6.90%

Lower error rates are denoted by green.

In Table 12 for QADI, the cosine similarity showed a mean of 46% for GPT 4, 43% for Bard (Gemini), and 37% for GPT 3.5. Table 13 exhibits a similar performance of 35% for GPT 4, 39% for Bard (Gemini), and 31% for GPT 3.5 on MADAR. This shows that GPT 4 is the best performer which aligns with the results of Alyafeai et al. (2023) and Peng et al. (2023). GPT 5 outperforms other models with a mean of 39% in MADAR. Noticeably, GPT 3.5 encountered the most struggles in translating to dialects from MSA which exhibits to a similar behavior in the conclusion drawn by Kadaoui et al. (2023).

On the other hand, sentence BERT shows the highest mean among all metrics as it uses a transformer model which makes it most accurate in finding similarities between the input dialect and the back-translated dialect. In addition, it showed consistent results for all LLMs across the two datasets. In Table 12 for QADI, Bard (Gemini) shows an average efficiency of 91%, hence outperforming GPT 4 and GPT 3.5 which shows an average efficiency of 89% and 87% consecutively. Similarly for MADAR in Table 13, Bard (Gemini) shows a total mean value of 93%, tying with GPT 3.5 whereas GPT 5 shows 92%, GPT 4 shows 90%. GPT 4 has witnessed a drop in accuracy due to poorer performance in LB dialect because of an outlier compared to other countries as its individual score shows 43% score, whereas others scored approximately 93%. This is due to an error occurred when running the data where sentences were translated to English instead of Arabic which drops the accuracy rate of the overall translation. Given that the error was only observed in the Lebanese dialect, it could be attributed that the model had unresolved difficulties in the background which was also passed down to the updated GPT 5 model as well.

In QADI dataset in Table 12, GPT 3.5 and Bard (Gemini) have an average score of 43% for ROUGE-L where GPT 4 scored an average of 47%. The analysis note that at least one Maghrebi dialect was of the highest ROUGE-L values observed for all models. However, GPT 3.5 achieved the top score for Palestine. This indicates a greater number of sentences overlap. These results indicate that GPT 4 was specifically well trained and consistent in at least one Magherbi dialect (e.g., Moroccon, Algerian, or Tunisian Arabic), whereas GPT 3.5 was a better fit in Palestinian dialect (i.e., Levantine Arabic).

**Figure 7:**
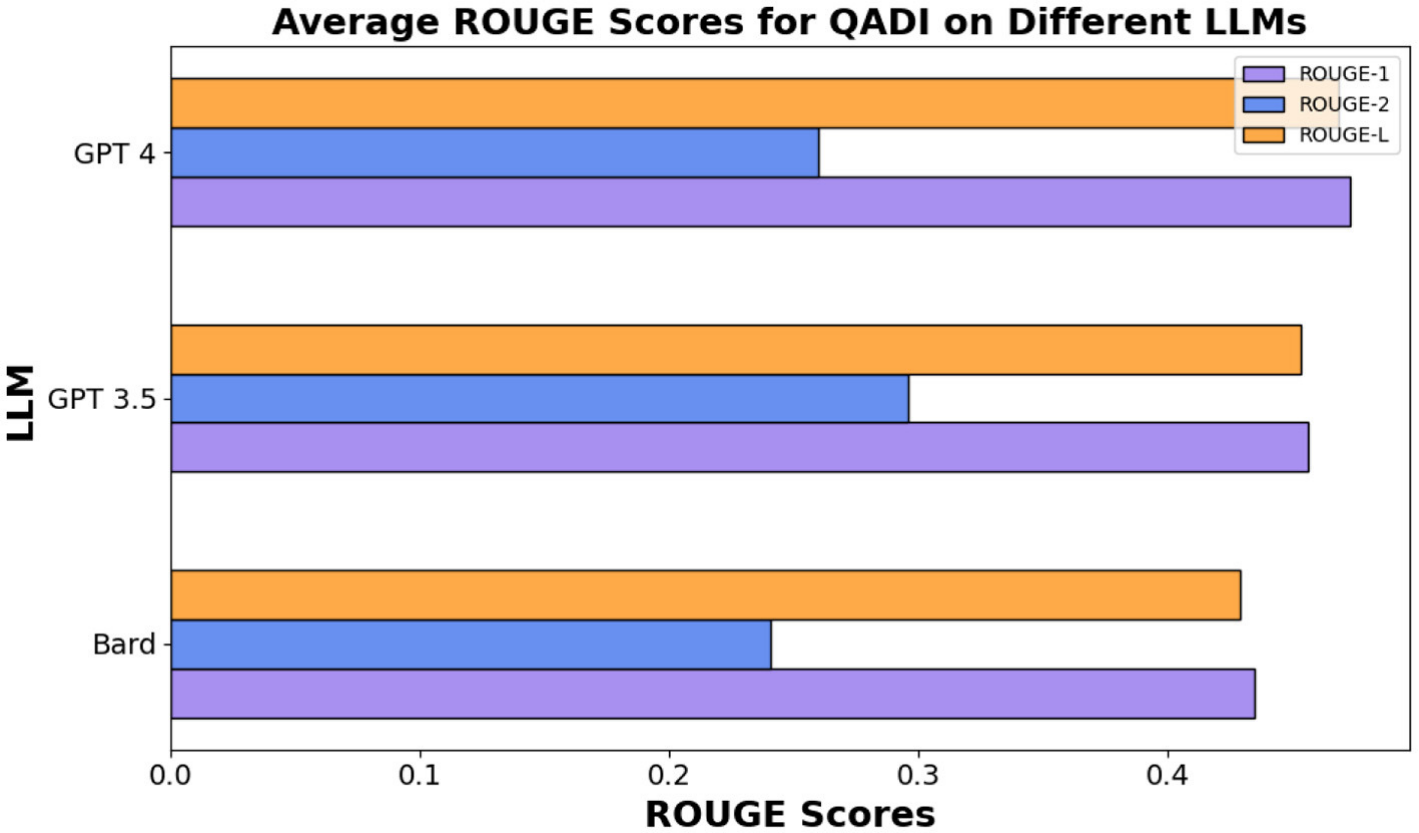
Average ROUGE scores for QADI dataset.

**Figure 8:**
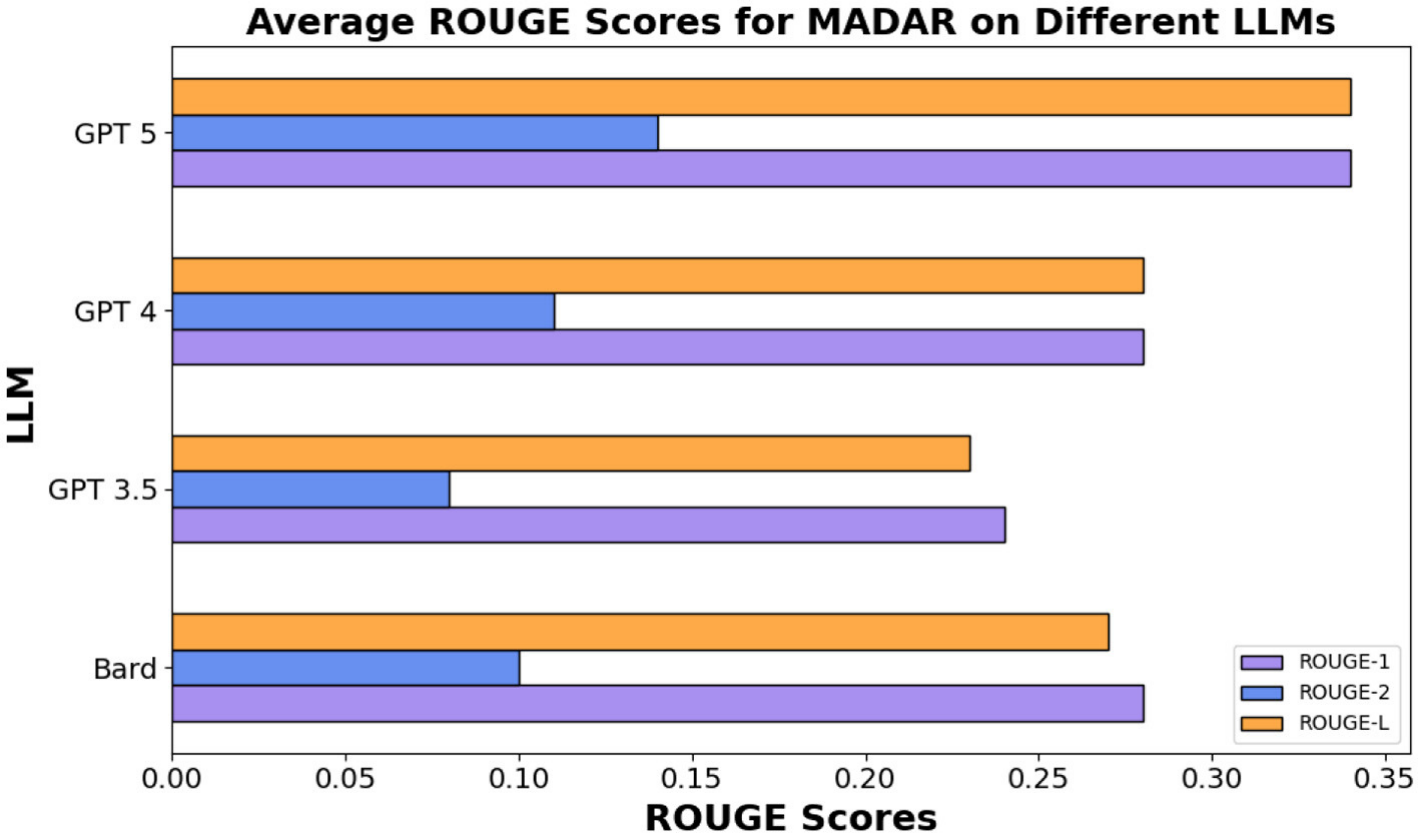
Average ROUGE scores for MADAR dataset.

In the same vein for the MADAR in Table 13, ROUGE-L scores were similar showing an average of 27%, 24%, 28% for Bard (Gemini), GPT 3.5/4, respectively, whereas GPT 5 outperforms other models showing 34%. Figures 7, 8 show the averages for each model to further illustrate the scores.

Overall, all three models among different datasets demonstrated a decently high average score for ROUGE-1 and ROUGE-L but lower scores for ROUGE-2. These results indicate that GPT 3.5, GPT 4, and Bard (Gemini) all had higher overlap between single words and long sequences between the compared text with GPT 4 being the highest in Figure 7, whereas GPT 5 clearly outperforms all other models in MADAR as demonstrated in Figure 8.

Overall, the results show that GPT 5 followed by GPT 4, Bard (Gemini), and GPT 3.5 are efficient in translating MSA to different DA, with slight difference and weaknesses noted in some of the dialects and models.

#### 4.2.2 TER

**Figure 9:**
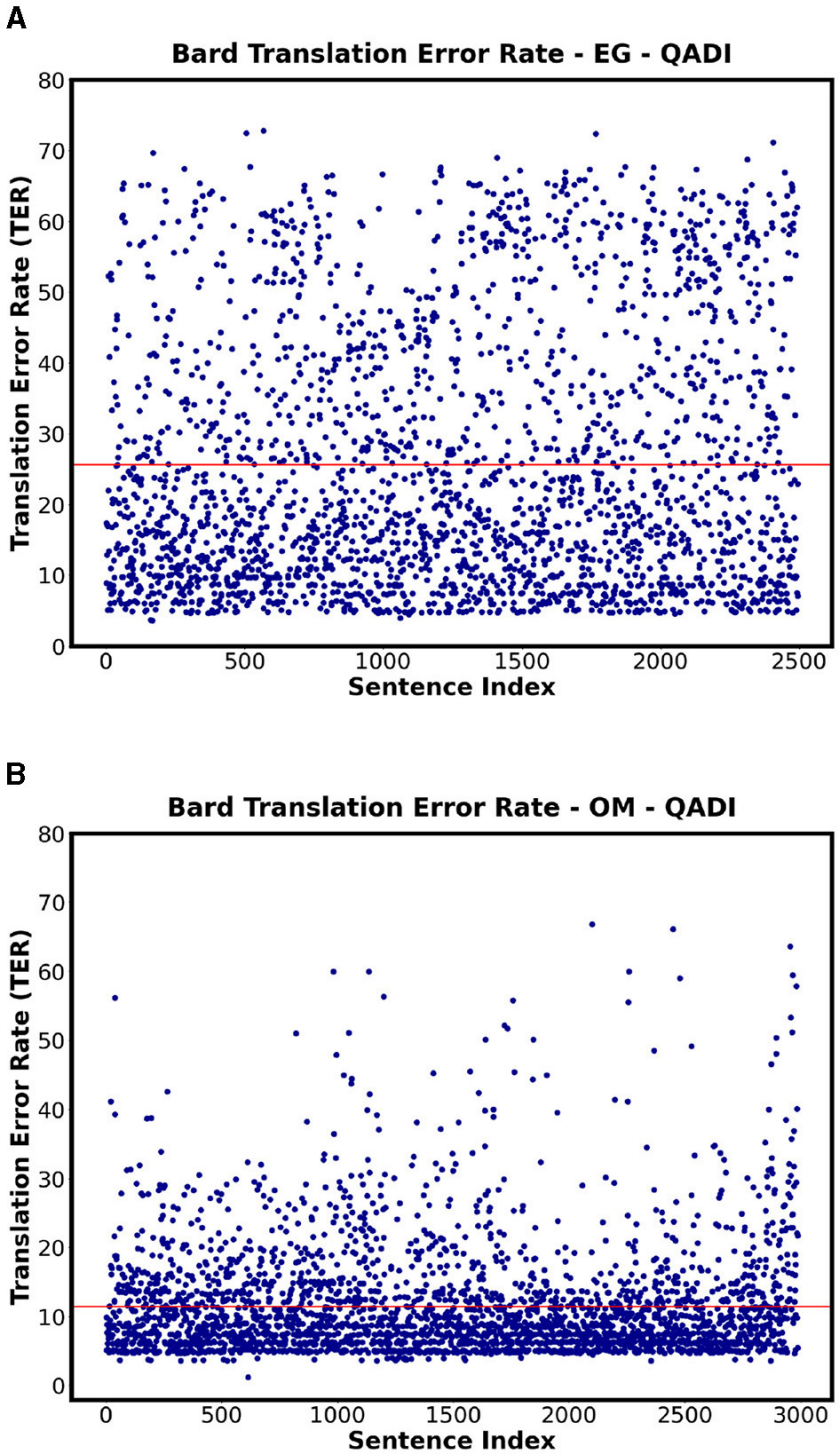
Scatter plots showing the TER for QADI datasets on Bard for highest and lowest countries. **(A)** Bard - EG Highest TER. **(B)** Bard - OM Lowest TER.

**Figure 10:**
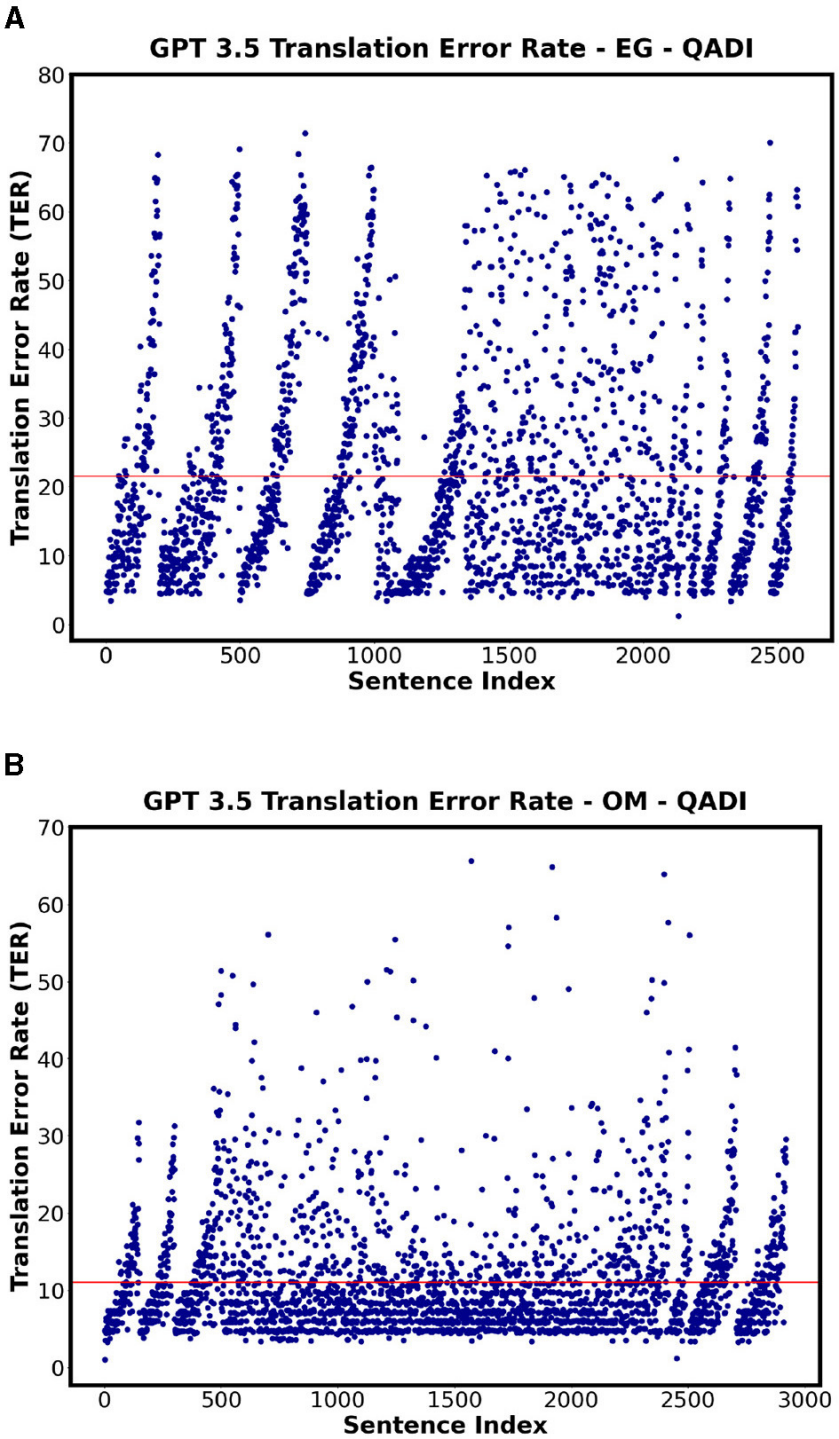
Scatter plots showing the TER for QADI datasets on GPT 3.5 for highest and lowest countries. **(A)** GPT 3.5 - EG Highest TER. **(B)** GPT 3.5 - OM Lowest TER.

Table 14 shows the TER for all the countries for QADI dataset for GPT 3.5, GPT 4, and MADAR, whereas the Figures 9, 10 visualize some dialects' results from QADI representing the average TER as a red line. The ranges of error demonstrated by TER range from approximately 10% up to 25% for all LLMs. Furthermore, the models have the lowest TER rate of approximately 11% for the OM dialect, whereas Bard (Gemini) has the highest worst TER rate in EG of 25.6%. Comparing the Gulf region countries (AE, BH, KW, OM, QA, and SA) specifically on GPT 3.5, OM showed the lowest TER of approximately 10%, whereas the other countries from the region showed an average ranging from 14% to 18%.

**Table 14:** TER for comparison for Bard, GPT 3.5, and GPT 4 for each dialect in the QADI dataset, where lower TER means higher performance.

**Dialect**	**Bard**	**GPT 3.5**	**GPT 4**
JO	18.08%	17.51%	18.02%
AE	17.02%	16.94%	17.75%
LB	18.16%	16.56%	17.34%
IQ	15.17%	15.06%	15.86%
BH	15.87%	14.97%	13.70%
DZ	16.64%	14.90%	13.37%
EG	25.60%	21.54%	22.91%
KW	14.81%	13.52%	12.47%
LY	18.65%	17.53%	17.66%
MA	14.80%	15.14%	17.23%
OM	11.43%	11.02%	10.82%
PL	11.82%	11.62%	11.38%
QA	17.98%	16.14%	14.83%
SA	15.89%	15.93%	16.75%
SD	19.10%	17.85%	16.89%
SY	14.59%	14.38%	14.42%
TN	16.28%	15.62%	16.69%
YE	16.04%	14.92%	15.35%

High error rates are colored by red, lower rates are denoted by green.

**Figure 11:**
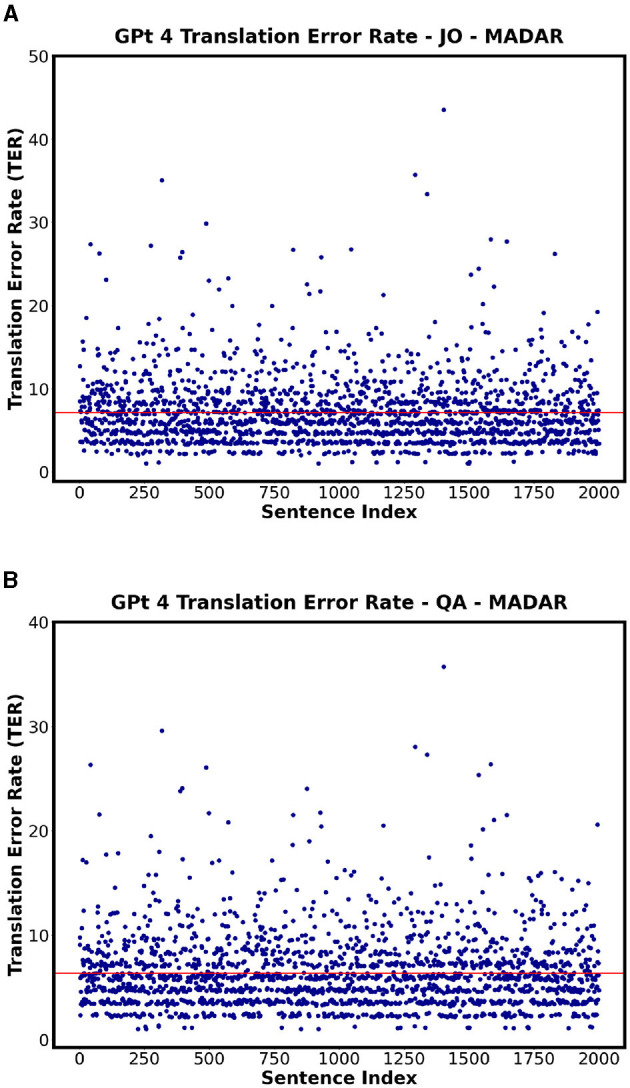
Scatter plots showing the TER for MADAR dataset on GPT 4 for each corresponding country. **(A)** GPT 4 - JO Highest TER. **(B)** GPT 4 - QA Lowest TER.

On the other hand, Table 15 and Figure 11 specifically showing GPT 4 illustrate the TER values of each country employing MADAR dataset as an example. In comparison with QADI dataset, the TER rates are closer together and have an overall lower value ranging from 6% to 7%, with JO being the highest and QA, SY, and OM being the lowest in the MADAR and QADI datasets. This may be explained by the fact that the MADAR dataset gathers sentences from a single source as a CORPUS, unlike the QADI dataset, which gathers sentences from X platform (Twitter) which is more prone to errors due to difficulty in filtering the sentences as tweets.

**Table 15:** TER Comparison for Bard, GPT 3.5, GPT 4, and GPT 5 for each dialect in the MADAR dataset, where lower TER means higher performance.

**Dialect**	**Bard**	**GPT 3.5**	**GPT 4**	**GPT 5**
JO	7.32%	7.11%	7.10%	6.95%
LB	6.54%	6.37%	6.36%	6.27%
IQ	6.66%	6.53%	6.49%	6.35%
DZ	7.14%	6.95%	6.93%	6.95%
EG	7.16%	7.02%	7.00%	6.88%
LY	7.06%	6.90%	6.89%	6.71%
MA	7.17%	7.10%	7.02%	6.88%
OM	7.20%	7.10%	7.04%	6.88%
PL	6.73%	6.57%	6.57%	6.41%
QA	6.49%	6.40%	6.35%	6.23%
SA	6.75%	6.61%	6.60%	6.50%
SD	7.14%	7.02%	7.03%	6.83%
SY	6.56%	6.38%	6.42%	6.30%
TN	6.71%	6.52%	6.53%	6.37%
YE	6.93%	6.75%	6.78%	6.61%

High error rates are colored by red, lower rates are denoted by green.

**Figure 12:**
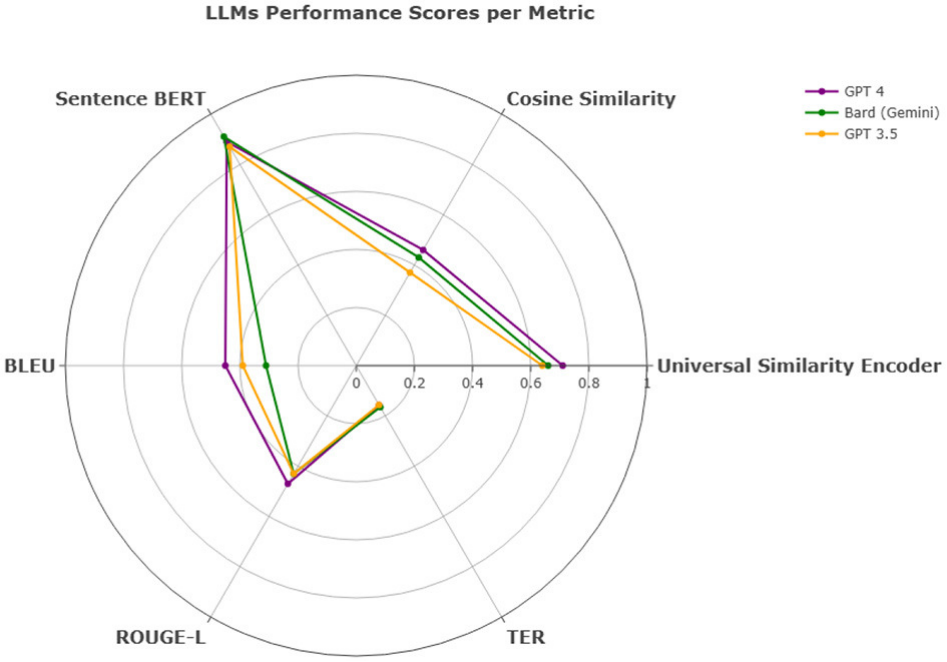
LLMs performance scores per metric - QADI dataset.

**Figure 13:**
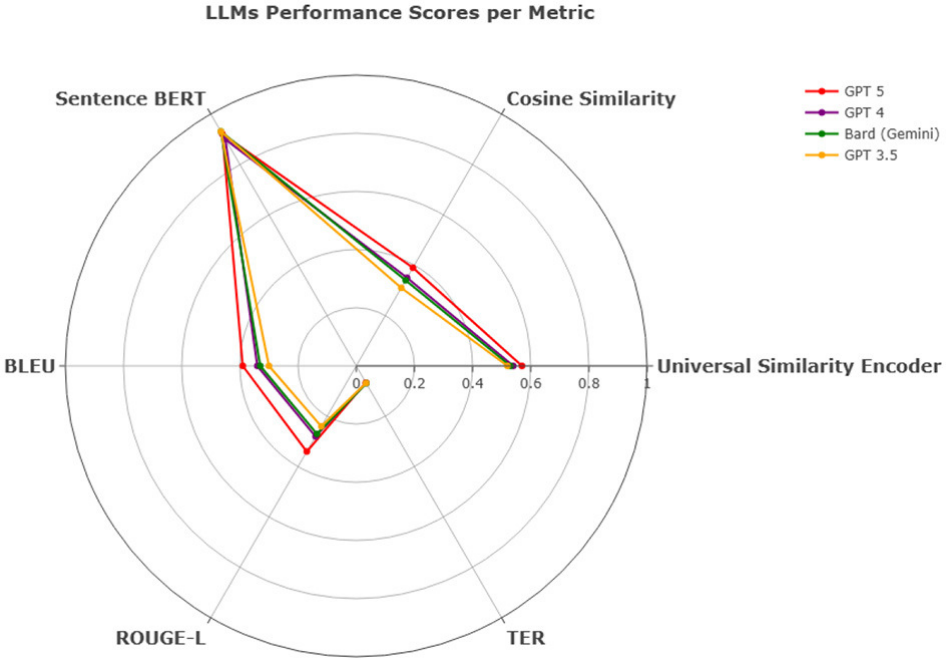
LLMs performance scores per metric - MADAR dataset.

Overall, in terms of efficiency and consistency combined, all models show competitive results and proved capable of translating multiple dialects regardless of the region as they all had approximately close values across the Middle East such as PL, LB, SY, and JO, the Gulf region such as KW, AE, SA, BH, OM, and QA, the Arab Maghreb region such as MA, LY, DZ, and TN and the African and Asian countries such as EG, SD, YE, and IQ. In QADI, GPT 4 outperforms the other LLMs in all similarity metrics and TER, Bard (Gemini) comes in the second place and then GPT 3.5 as shown in Table 12 whereas GPT 5 outperforms GPT 4 and other models in MADAR in Table 13 proving it being a more reliable model in translating from MSA to DA. This is further demonstrated in Figures 12, 13 which further demonstrate LLM performance upon the metrics used in this study. Models exhibited consistent scores among all metrics with GPT 5 being the highest and most appropriate LLM to deal with Arabic dialects.

#### 4.2.3 ANOVA

ANOVA test is a common test used to check whether the data and mean difference are significant based on different conditions and factors. In previous sections, we found that the average translation performance among similarity metrics and TER are quite similar. To better understand the significance difference, one-way ANOVA is applied to all countries and models with alpha 0.05 threshold. We have applied Shapiro–Wilk test diagnostic to verify the residuals normality and applicable for ANOVA. This is a similar approach adapted by Alabdullah et al. (2025). The ANOVA results are shown in Table 16 for QADI and Table 17 for the MADAR dataset. The models GPT and Gemini are the independent variables and the performance metrics including similarity metrics, BLEU, and ROUGE were considered dependent variables. In reference to Table 16, ANOVA test is applied among all similarity metrics, and there is a significant difference between the model translation performance with a *p*-value close to 0 in universal similarity encoder, cosine similarity, and sentence BERT, which indicates that the probability of the average similarities are different is approximately 99.96%. Metrics such as BLEU, ROUGE-L, and TER show insignificant difference among the models meaning that all models have similar scores/error rates in translation. Moreover, the f-value < 1 suggested that there is no variance across the means.

**Table 16:** ANOVA results for models per metric - QADI dataset.

**Metric**	***p*-value**	***F*-statistic**
Universal similarity encoder	0.009111	7.65
Cosine similarity	0.000006	28.85
Sentence BERT	0.000068	20.57
BLEU	0.058	3.85
ROUGE-L	0.00018	0.16
TER	0.56	0.59

**Table 17:** ANOVA results for models per metric - MADAR dataset.

**Metric**	***p-*value**	***F*-statistic**
Universal similarity encoder	0.005	4.64
Cosine similarity	0.00009	8.57
Sentence BERT	0.44	0.91
BLEU	0.000029	9.73
ROUGE-L	0.68	7.87
TER	0.31	1.2

As for MADAR, Table 17 shows that there is no difference between the means and all models exhibited similar translation performance on sentence BERT, ROUGE-L, and TER. However, the other metrics show significant differences between the LLMs' scores.

#### 4.2.4 Evaluation divergence (lexical vs. semantic metrics)

Upon evaluating different models with different performance metrics, some conflicts between the metrics were noted. To strengthen our analysis, we have chosen different metrics, each evaluating a certain category of the LLMs ability. BLEU and ROUGE rely on lexical overlap with the reference translation (the original dialect in our case) and count the n-gram overlap. On the other hand, universal similarity encoder and sentence-BERT are semantic measures that focus on meaning equivalence regardless of literal word matching. TER is concerned with the number of edits to match the generated dialect with the base dialect reference. As we are evaluating the 15 dialects, this variation often involves synonym choice, morphological difference, and substitutions. A model can semantically translate to the correct dialect yet not the exact word matching which leads to lower BLEU and ROUGE scores. Conversely, high lexical overlap does not always guarantee semantic accuracy if the matched words are used in a different sense. The is noted in Table 9, and some dialects such as DZ and EG scored low BLEU/ROUGE scores while achieving high values in the semantic evaluation perspective. These findings support our approach and analysis, highlighting the need to adapt different metric scores, as each captures different aspects of LLM translation quality.

### 4.3 Effects of model accuracy

#### 4.3.1 Few-shots analysis

In this section, we will explore the opportunity to check whether increasing the prompt size from zero-shot to few-shot would enhance the translation quality of each LLM. We used the MADAR dataset as it has more consistency in results with TN having the lowest similarity scores in Table 18 and a high TER rate as shown in Table 19, indicating a need to enhance the translation quality of this dialect. In both datasets, the models showed the least translation performance for the Tunisian dialect, and this is correspondence to Sallam and Mousa (2024) research as well. QADI showed inconsistency in similarity scores. Which could be attributed to the fact that QADI gathers its sentences from X platform, which means that although the sentences are gathered from the same geolocation, this does not mean that they all belong to the same dialect.

**Table 18:** Countries with lowest values in MADAR dataset similarity metrics.

**Model**	**Univ. Sim. Enc**.	**Cosine Sim**.	**Sent. BERT**	**BLEU**	**ROUGE**
Bard	TN	TN	PL	TN	TN
GPT 3.5	TN	TN	LB	TN	TN
GPT 4	MA but TN similar score	TN–MA	LB	TN–MA	TN
GPT 5	DZ	DZ	Not applicable	DZ	DZ

**Table 19:** Countries with highest TER values in MADAR dataset.

**Model**	**TER**
Bard	JO but TN similar score
GPT 3.5	JO but TN similar score
GPT 4	JO but TN similar score
GPT 5	JO but DZ similar score

Although adding a few-shot approach provides models with additional examples and reference points, most models exhibited a decline performance in compared to zero-shot. This is illustrated in Tables 20, 21. In particular, GPT 3.5 showed consistency, with no significant differences between the zero-shot and few-shot approach. Suddenly, GPT 4 translated almost 35% of the input sentences into English despite clear instructions. This might be explained by the model's biases or training to adapt English translations in unclear contexts for the model. Given that the few-shot prompt is considered as a long prompt and has several examples and details, GPT 4 might find the prompt ambiguous and refer to the default language setting, which is “English”.

**Table 20:** Tunisia zero-shot metric performance.

**Model**	**USE**	**Cosine Sim**	**S-BERT**	**BLEU**	**Rouge-L**	**TER**
Bard	0.48	0.26	0.93	0.25	0.41	6.71%
GPT 3.5	0.48	0.24	0.93	0.23	0.49	6.52%
GPT 4	0.48	0.26	0.93	0.25	0.45	6.53%

**Table 21:** Tunisia few-shot metric performance.

**Model**	**USE**	**Cosine Sim**	**S-BERT**	**BLEU**	**Rouge-L**	**TER**
Bard	0.47	0.23	0.93	0.21	0.15	6.77%
GPT 3.5	0.48	0.24	0.92	0.24	0.16	6.53%
GPT 4	0.32	0.20	0.93	0.20	0.12	6.64%

#### 4.3.2 Impact of sentence length on model accuracy

This subsection analyzes the impact of sentence length on translation accuracy, hence addressing the third research question. Since the universal similarity encoder is used to compare two sentences, it enabled us to explore the correlation.

**Figure 14:**
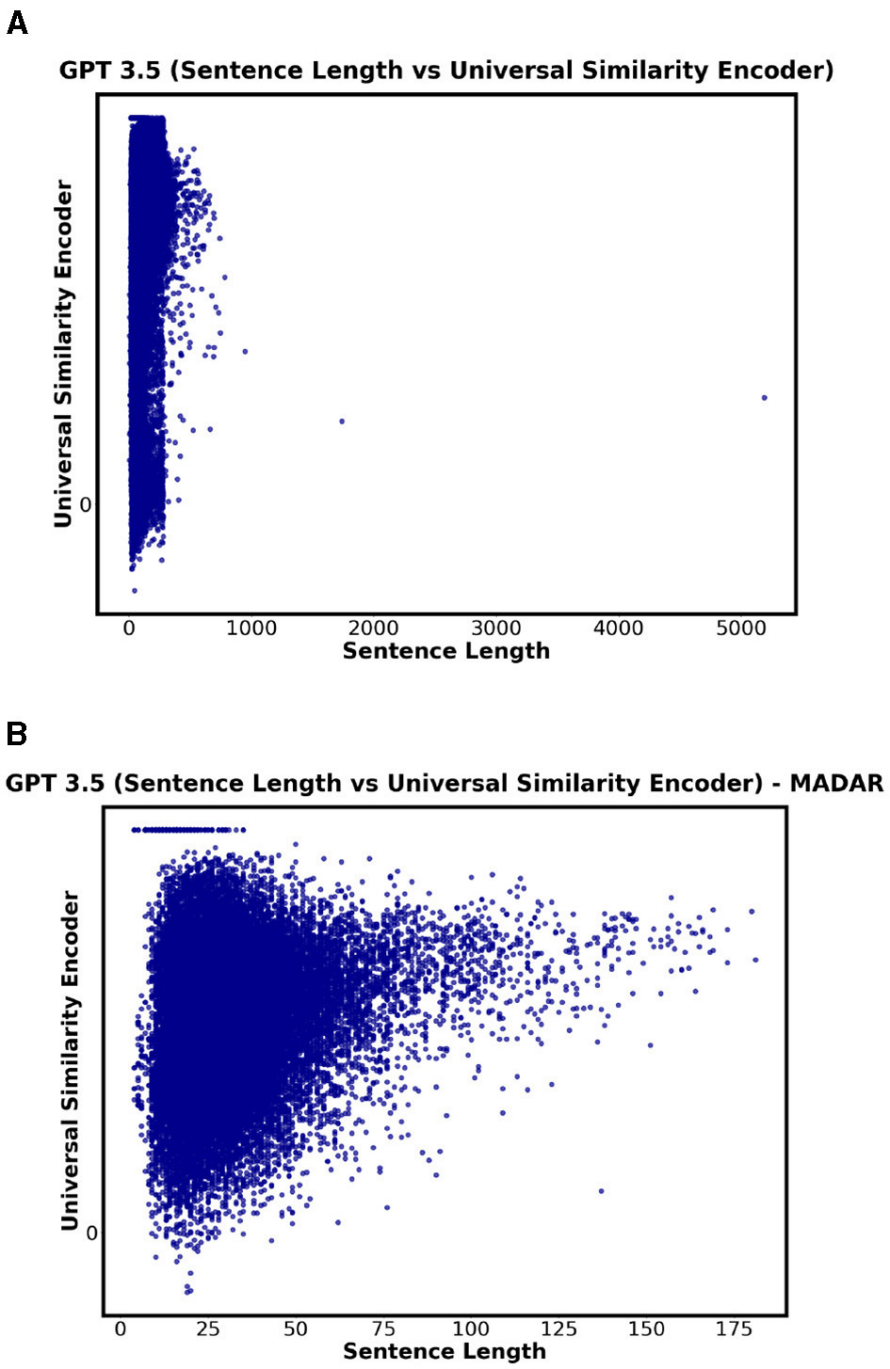
Correlation (sentence length vs universal similarity) for GPT 3.5. **(A)** GPT 3.5 - QADI. **(B)** GPT 3.5 - MADAR.

For QADI dataset, the highest correlation was 0.42 in MA for GPT 4. The highest correlation for Bard was 0.39 in QA. GPT 3.5 showed a low correlation between the sentence length and the translation accuracy (i.e., similarity between input and output). Figure 14 visualizes the results where showing no strong correlation between the sentence length and the universal similarity encoder. Such low positive correlations indicate that there is no relation between the sentence length and the accuracy of the translation.

For MADAR, GPT 3.5/4 show a weak correlation, yet the highest compared to Bard with a value of 0.24 for some Maghreb Countries (i.e., DZ, MA, and TN) where Bard show no significant correlation. Figure 14 supports this finding as GPT 3.5/4 indicate a broader range of similarity scores as sentence length varies.

## 5 Conclusion

### 5.1 Concluding remarks

The study utilizes the QADI and MADAR datasets to evaluate the performance of GPT 3.5, GPT 4, and Bard (Gemini) in translating MSA to Arabic dialects, with GPT 5 evaluated exclusively on the MADAR dataset. Several performance metrics such as cosine similarity, universal similarity encoder, sentence BERT, BLEU, ROUGE, and TER were used to test the models' efficiency and accuracy. The analysis revealed close translations among LLMs in similarity and error rate. In QADI dataset, there was a significant difference between the models where GPT 4 was the best LLM in translating MSA to Arabic dialects showing a *p*-value of 0.000006 through ANOVA test on cosine similarity metric. It shows significant difference on all metrics except for BLEU and TER. For the MADAR dataset, there were no significant differences in translation performance measuring on sentence BERT, ROUGE-L, and TER. However, the results show significant differences through universal similarity encoder, cosine similarity, and BLEU, with GPT 5 being the top performer. GPT 4 demonstrates the best performance across both datasets (MADAR and QADI); it consistently showed high translation quality with low error rates. This proves the models sufficiency and the ability to be used in several dialect contexts and applications. GPT-4 showed consistent high translation scores for the majority of metrics, specifically on Levantine and Egyptian dialects; however, it shows low results on Maghrebi regions such as Tunisian dialect. Overall, GPT-4 provides the most reliable performance while GPT 5 outperforms all models specifically on the MADAR dataset in finding sentences overlap measured by BLEU and ROUGE-L.

However, its performance is not uniform across all dialects' while it excels in dialects with larger training representation (e.g., Egyptian and Levantine), the accuracy slightly decreases in underrepresented dialects (e.g., Maghrebi). On the MADAR dataset, GPT-5 shows particularly strong performance on overlap-sensitive metrics such as BLEU and ROUGE-L, suggesting it captures sentence-level correspondences more effectively. Taken together, GPT-4 provides the most reliable overall performance across both datasets, while GPT-5 demonstrates an emerging advantage in fine-grained similarity for MADAR dialectal translations.

Furthermore, models have shown TER rates ranging from 6% up to 25%, indicating that despite slight errors, their translations are generally considered to be of good quality. However, GPT has shown better response to a given prompt in terms of output results compared to Bard (Gemini). GPT in all versions specifically GPT 5 showed the best results for translation through the Levant countries. Zero-shot prompts were adapted for all countries, while few-shot for the country with the least translation performance, Tunisia. Unexpectedly, the few-shot technique did not enhance the performance of translation especially for Bard (Gemini) and GPT 4 as they performed worse while GPT 3.5 performed consistently in both prompting techniques. Overall, all LLMs proved capable and efficient in translating diverse Arabic dialects from over 15 countries to provide valuable insights for future applications in NLP.

This research establishes a benchmark for Arabic dialect translation and derives significant findings for advancing NLP capabilities in Arabic, paving the way for more inclusive and efficient models that address the linguistic diversity of the Arab world. Other researchers in the field may rely on GPT 4 and GPT 5 over GPT 3.5 and adapt Bard (Gemini), considering them feasible and effective LLMs for handling underrepresented languages, particularly Arabic and its linguistic complexities. The study also opens opportunities for future work, such as incorporating open source models, improving data sets, and optimizing prompting techniques. Moreover, we show the impact of few-shot prompting and how its impact was not significant, which could be replaced by other alternatives or prompt engineering techniques in future or relevant works.

### 5.2 Future works

We are aiming to extend this research by incorporating additional Arabic LLMs and other well-known applicable LLMs to generalize our findings. In addition, more data samples and datasets can be included to strengthen the analysis. Looking ahead, enhancing prompt and prompting techniques to optimize the translation process would add value to this research.

### 5.3 Limitations

This study faces several limitations that could influence the study results. Despite their remarkable success in various NLP tasks and the popularity of closed-source LLMs, models such as GPT 3.5, GPT 4, and GPT 5 have several limitations (Yu et al., 2023). These models are accessed through APIs which eliminates the need for computer infrastructure. Although cloud-based AI services are easy to use, they lack control over processing or training data. Furthermore, it is challenging to produce studies on closed-source models due to the high expense of conducting experiments through APIs. Another limitation is that the LLMs are closed models, as the name suggests, closed LLMs lack transparency in their internal architecture and training process, making it difficult for researchers to fully understand the output generation. The limitations also include cost constraints while running LLMs such as GPT 3.5/4 and Bard (Gemini) which results in running only 50K out of 500K samples in QADI dataset. Expanding the sample size in future studies could improve the robustness and reliability of the results. Moreover, both GPT and Bard (Gemini) had restrictions on the rate limit (i.e., the number of API requests). Thus, limiting the running process of the data to a specific rate daily, this consumed the time to complete the running. It is possible that recently published versions have increased the rate limit, which could be explored. In addition, there is lack in LLMs that can deal with Arabic dialects; having more LLMs would definitely strengthen the comparison. While this study adapted datasets encompassing 15 to 18 dialects, it does not cover all 22 Arabic-speaking countries, thus limiting the generalizability of the findings. Furthermore, QADI dataset, which is collected from X, may require cleaning to remove slang and informal expressions in social media, which can improve the quality of translation outputs. In addition, only one dataset (i.e., MADAR) had a MSA baseline, which was substituted by a back-translation algorithm for the QADI dataset. This approach may potentially limit the accuracy and effectiveness of the translations derived from QADI dataset. Moreover, the MADAR dataset exhibits a travel domain bias, which may affect the findings and limit the model's translation capability to other domains. In some cases, the models were not able to translate the dialect, resulting in an empty output, English translated sentence instead of Arabic or incomplete response. Finally, since most of the metrics are calculated as mean scores with only a single inferential statistical test (ANOVA) applied, generalizing the results might be tricky.

## Data Availability

The raw data supporting the conclusions of this article will be made available by the authors, without undue reservation.

## References

[B1] Abdelali A. Mubarak H. Samih Y. Hassan S. Darwish K. (2020). Arabic dialect identification in the wild. arXiv preprint arXiv:2005.06557.32490098

[B2] Abdelaziz A. A. A. Elneima A. H. Darwish K. (2024). “LLM-based MT data creation: Dialectal to MSA translation shared task,” in Proceedings of the 6th Workshop on Open-Source Arabic Corpora and Processing Tools (OSACT) with Shared Tasks on Arabic LLMs Hallucination and Dialect to MSA Machine Translation@ LREC-COLING 2024, 112–116.

[B3] Abu-Haidar F. (2011). “Shifting boundaries: the effect of msa on dialect convergence in Baghdad,” in Perspectives on Arabic Linguistics: Papers from the Annual Symposium on Arabic Linguistics. Volume IV: Detroit, Michigan 1990 (John Benjamins Publishing Company), 91–106. 10.1075/cilt.85.07abu

[B4] Alabdullah A. Han L. Lin C. (2025). Advancing dialectal Arabic to modern standard Arabic machine translation. arXiv preprint arXiv:2507.20301.

[B5] Alahmari S. Atwell E. Saadany H. (2024). “Sirius_Translators at OSACT6 2024 shared task: fin-tuning ara-T5 models for translating arabic dialectal text to modern standard Arabic,” in Proceedings of the 6th Workshop on Open-Source Arabic Corpora and Processing Tools (OSACT) with Shared Tasks on Arabic LLMs Hallucination and Dialect to MSA Machine Translation@ LREC-COLING 2024 (ELRA and ICCL), 117–123.

[B6] Al-Gaphari G. Al-Yadoumi M. (2010). A method to convert Sana'ani accent to modern standard Arabic. Int. J. Inf. Sci. Manag. 8, 39–49.

[B7] Alimi T. Boujelbene R. Derouich W. Belguith L. (2024). Fine-tuned transformers for translating multi-dialect texts to modern standard Arabic. Int. J. Cogn. Lang. Sci. 18, 679–684.

[B8] Allingham J. U. Ren J. Dusenberry M. W. Gu X. Cui Y. Tran D.. (2023). “A simple zero-shot prompt weighting technique to improve prompt ensembling in text-image models,” in International Conference on Machine Learning (PMLR), 547–568.

[B9] Alyafeai Z. Alshaibani M. S. AlKhamissi B. Luqman H. Alareqi E. Fadel A. (2023). Taqyim: evaluating Arabic NLP tasks using ChatGPT models. arXiv preprint arXiv:2306.16322.

[B10] Atwany H. Rabih N. Mohammed I. Waheed A. Raj B. (2024). “OSACT 2024 task 2: Arabic dialect to MSA translation,” in Proceedings of the 6th Workshop on Open-Source Arabic Corpora and Processing Tools (OSACT) with Shared Tasks on Arabic LLMs Hallucination and Dialect to MSA Machine Translation@ LREC-COLING 2024, 98–103.

[B11] Baert G. Gahbiche S. Gadek G. Pauchet A. (2020). “Arabizi language models for sentiment analysis,” in Proceedings of the 28th International Conference on Computational Linguistics, 592–603. 10.18653/v1/2020.coling-main.5136568019

[B12] Bakr H. A. Shaalan K. Ziedan I. (2008). “A hybrid approach for converting written egyptian colloquial dialect into diacritized Arabic,” in The 6th International Conference on Informatics and Systems, infos2008. Cairo University (Citeseer).

[B13] Baniata L. H. Kang S. Ampomah I. K. E. (2022). A reverse positional encoding multi-head attention-based neural machine translation model for Arabic dialects. Mathematics 10:3666. 10.3390/math10193666

[B14] Baniata L. H. Park S. Park S.-B. (2018). A neural machine translation model for Arabic dialects that utilizes multitask learning (MTL). Comput. Intell. Neurosci. 2018:7534712. 10.1155/2018/7534712 30643518 PMC6311304

[B15] Behr D. (2017). Assessing the use of back translation: the shortcomings of back translation as a quality testing method. Int. J. Soc. Res. Methodol. 20, 573–584. 10.1080/13645579.2016.125218835289191

[B16] Bhat S. Varma V. Pedanekar N. (2023). “Generative models for indic languages: evaluating content generation capabilities,” in Proceedings of the 14th International Conference on Recent Advances in Natural Language Processing, 187–195. 10.26615/978-954-452-092-2_021

[B17] Bouamor H. Habash N. Oflazer K. (2014). “A multidialectal parallel corpus of Arabic,” in Proceedings of the Ninth International Conference on Language Resources and Evaluation (LREC'14), Reykjavik, Iceland (European Language Resources Association (ELRA)), 1240–1245.

[B18] Bouamor H. Hassan S. Habash N. (2019). “The MADAR shared task on Arabic fine-grained dialect identification,” in Proceedings of the Fourth Arabic Natural Language Processing Workshop, 199–207. 10.18653/v1/W19-462236568019

[B19] Chan V. Tang W. K.-W. (2024). “GPT and translation: a systematic review,” in 2024 International Symposium on Educational Technology (ISET) (IEEE), 59–63. 10.1109/ISET61814.2024.00021

[B20] Chang Y. Wang X. Wang J. Wu Y. Yang L. Zhu K.. (2023). A survey on evaluation of large language models. ACM Trans. Intell. Syst. Technol. 15, 1–45. 10.1145/3641289

[B21] De Varda A. Marelli M. (2023). “Scaling in cognitive modelling: a multilingual approach to human reading times,” in Proceedings of the 61st Annual Meeting of the Association for Computational Linguistics (Volume 2: Short Papers), 139–149. 10.18653/v1/2023.acl-short.1436568019

[B22] Demidova A. Atwany H. Rabih N. Sha'ban S. (2024). “Arabic train at NADI 2024 shared task: LLMs' ability to translate Arabic dialects into Modern Standard Arabic,” in Proceedings of the Second Arabic Natural Language Processing Conference (Bangkok, Thailand: Association for Computational Linguistics). 10.18653/v1/2024.arabicnlp-1.80

[B23] Devlin J. Chang M.-W. Lee K. Toutanova K. (2018). BERT: pre-training of deep bidirectional transformers for language understanding. arXiv preprint arXiv:1810.04805.

[B24] Eberhard D. Simons G. Fennig C. (2019). Ethnologue: Languages of Asia, Twenty-Second Edition. Ethnologue Series. Sil International, Global Publishing.

[B25] Guellil I. Azouaou F. Abbas M. (2017). “Neural vs. statistical translation of Algerian Arabic dialect written with Arabizi and Arabic letter,” in The 31st Pacific Asia Conference on Language, Information and Computation Paclic.

[B26] Guellil I. Saâdane H. Azouaou F. Gueni B. Nouvel D. (2021). Arabic natural language processing: an overview. J. King Saud Univ. Comput. Inf. Sci. 33, 497–507. 10.1016/j.jksuci.2019.02.006

[B27] Hadi M. U. al tashi Qureshi, R. Shah A. Irfan M. Zafar A. Shaikh M. B.. (2023). Large language models: a comprehensive survey of its applications, challenges, limitations, and future prospects. Authorea Preprints 1, 1–26. 10.36227/techrxiv.23589741.v2

[B28] Hamada S. Marzouk R. M. (2018). Developing a Transfer-Based System for Arabic Dialects Translation. Cham: Springer International Publishing, 121–138. 10.1007/978-3-319-67056-0_7

[B29] Hamed M. M. Hreden M. Hennara K. Aldallal Z. Chrouf S. AlModhayan S. (2025). “Lahjawi: Arabic cross-dialect translator,” in Proceedings of the 4th Workshop on Arabic Corpus Linguistics (WACL-4), 12–24.

[B30] Harrat S. Meftouh K. Smaili K. (2019). Machine translation for Arabic dialects (survey). Inf. Proc. Manag. 56, 262–273. 10.1016/j.ipm.2017.08.00340711496

[B31] Jiang E. Olson K. Toh E. Molina A. Donsbach A. Terry M.. (2022). “Promptmaker: prompt-based prototyping with large language models,” in CHI Conference on Human Factors in Computing Systems Extended Abstracts, 1–8. 10.1145/3491101.3503564

[B32] Jiang Z. Xu F. F. Araki J. Neubig G. (2020). How can we know what language models know? Trans. Assoc. Comput. Linguist. 8, 423–438. 10.1162/tacl_a_00324

[B33] Jibrin F. Mughaus R. Abudalfa S. Ahmed M. Abdelali A. (2025). An empirical evaluation of Arabic text formality transfer: a comparative study. Lang. Resour. Evaluat. 2024, 1–16.

[B34] Joshi A. Dabre R. Kanojia D. Li Z. Zhan H. Haffari G.. (2024). Natural language processing for dialects of a language: a survey. arXiv preprint arXiv:2401.05632.

[B35] Kadaoui K. Magdy S. M. Waheed A. Khondaker M. T. I. El-Shangiti A. O. Nagoudi E. M. B.. (2023). TARJAMAT: evaluation of bard and chatGPT on machine translation of ten Arabic varieties. arXiv preprint arXiv:2308.03051.

[B36] Kasneci E. Sessler K. Küchemann S. Bannert M. Dementieva D. Fischer F.. (2023). ChatGPT for good? On opportunities and challenges of large language models for education. Learn. Indiv. Differ. 103:102274. 10.1016/j.lindif.2023.10227438266214

[B37] Keselman H. J. Huberty C. J. Lix L. M. Olejnik S. Cribbie R. A. Donahue B.. (1998). Statistical practices of educational researchers: an analysis of their ANOVA, MANOVA, and ANCOVA analyses. Rev. Educ. Res. 68, 350–386. 10.3102/0034654306800335038293548

[B38] Kheiri K. Karimi H. (2023). SentimentGPT: exploiting GPT for advanced sentiment analysis and its departure from current machine learning. arXiv preprint arXiv:2307.10234.

[B39] Khered A. S. Benkhedda Y. Batista-Navarro R. T. (2025). “Dial2MSA-verified: a multi-dialect arabic social media dataset for neural machine translation to modern standard Arabic,” in Proceedings of the 4th Workshop on Arabic Corpus Linguistics (WACL-4), 50–62.

[B40] Khondaker M. T. I. Waheed A. Nagoudi E. M. B. Abdul-Mageed M. (2023). GPTAraEval: a comprehensive evaluation of ChatGPT on Arabic NLP. arXiv preprint arXiv:2305.14976.

[B41] Khoshafah F. (2023). ChatGPT for Arabic-English translation: Evaluating the accuracy. Europe PubMed Central (PMC) Repository. 10.21203/rs.3.rs-2814154/v1

[B42] Koubaa A. Ammar A. Ghouti L. Najar O. Sibaee S. (2024). ArabianGPT: Native Arabic GPT-based Large Language. arXiv preprint arXiv:2402.15313.

[B43] Lilli S. (2023). ChatGPT-4 and Italian dialects: assessing linguistic competence. Umanistica Digitale 16, 235–263.

[B44] Lin C.-Y. Hovy E. (2003). “Automatic evaluation of summaries using N-gram co-occurrence statistics,” in Proceedings of the 2003 Human Language Technology Conference of the North American Chapter of the Association for Computational Linguistics, 150–157. 10.3115/1073445.107346539493181

[B45] López Espejel J. Ettifouri E. H. Yahaya Alassan M. S. Chouham E. M. Dahhane W. (2023). GPT-3.5, GPT-4, or BARD? Evaluating LLMs reasoning ability in zero-shot setting and performance boosting through prompts. Nat. Lang. Proc. J. 5:100032. 10.1016/j.nlp.2023.100032

[B46] Malaysha S. El-Haj M. Ezzini S. Khalilia M. Jarrar M. Almujaiwel S.. (2024). AraFinNlp 2024: The first Arabic financial NLP shared task. arXiv preprint arXiv:2407.09818.

[B47] Mashaabi M. Al-Khalifa S. Al-Khalifa H. (2024). A survey of large language models for Arabic language and its dialectss. arXiv preprint arXiv:2410.20238.

[B48] Mohamed E. Mohit B. Oflazer K. (2012). “Transforming standard arabic to colloquial Arabic,” in Proceedings of the 50th Annual Meeting of the Association for Computational Linguistics (Volume 2: Short Papers), 176–180.

[B49] Mrinalini K. Vijayalakshmi P. Nagarajan T. (2022). SBSim: a sentence-BERT similarity-based evaluation metric for indian language neural machine translation systems. IEEE/ACM Trans. Audio, Speech, Lang. Proc. 30, 1396–1406. 10.1109/TASLP.2022.3161160

[B50] Okpor M. D. (2014). Machine translation approaches: issues and challenges. Int. J. Comput. Sci. Issues 11:159.

[B51] Papineni K. Roukos S. Ward T. Zhu W.-J. (2002). “BLEU: a method for automatic evaluation of machine translation,” in Proceedings of the 40th annual meeting of the Association for Computational Linguistics, 311–318. 10.3115/1073083.1073135

[B52] Penedo G. Malartic Q. Hesslow D. Cojocaru R. Cappelli A. Alobeidli H.. (2023). The RefinedWeb dataset for falcon LLM: outperforming curated corpora with web data only. arXiv preprint arXiv:2306.01116.

[B53] Peng K. Ding L. Zhong Q. Shen L. Liu X. Zhang M.. (2023). Towards making the most of ChatGPT for machine translation. arXiv preprint arXiv:2303.13780.

[B54] Ridouane T. Bouzoubaa K. (2014). “A hybrid approach to translate Moroccan Arabic dialect,” in 2014 9th International Conference on Intelligent Systems: Theories and Applications (SITA-14) (IEEE), 1–5.

[B55] Sallam M. Mousa D. (2024). Evaluating ChatGPT performance in Arabic dialects: a comparative study showing defects in responding to Jordanian and Tunisian general health prompts. Mesopot. J. Artif. Intell. Healthcare. 2024, 1–7. 10.58496/MJAIH/2024/001

[B56] Salloum W. Habash N. (2012). “Elissa: a dialectal to standard Arabic machine translation system,” in Proceedings of COLING 2012: Demonstration Papers, 385–392.

[B57] Sghaier M. A. Zrigui M. (2020). Rule-based machine translation from Tunisian dialect to modern standard Arabic. Procedia Comput. Sci. 176, 310–319. 10.1016/j.procs.2020.08.033

[B58] Shaikh S. Daudpota S. M. Yayilgan S. Y. Sindhu S. (2023). “Exploring the potential of large-language models (LLMs) for student feedback sentiment analysis,” in 2023 International Conference on Frontiers of Information Technology (FIT) (IEEE), 214–219. 10.1109/FIT60620.2023.00047

[B59] Sibaee S. Nacar O. Al-Habashi Y. Ammar A. Boulila W. (2025). SHAMI-MT: a Syrian Arabic dialect to modern standard Arabic bidirectional machine translation system. arXiv preprint arXiv:2508.02268.

[B60] Steele J. L. (2023). To GPT or not GPT? Empowering our students to learn with AI. Comput. Educ. 5:100160. 10.1016/j.caeai.2023.100160

[B61] Wright W. Caspari C. P. (2011). A Grammar of the Arabic Language. Cosimo, Inc.

[B62] Yong Z.-X. Schoelkopf H. Muennighoff N. Aji A. F. Adelani D. I. Almubarak K.. (2022). BLOOM+1: adding language support to BLOOM for zero-shot prompting. arXiv preprint arXiv:2212.09535.

[B63] Yu H. Yang Z. Pelrine K. Godbout J. F. Rabbany R. (2023). Open, closed, or small language models for text classification? *arXiv preprint arXiv:2308.10092*.

[B64] Zhang L. Fan H. Peng C. Rao G. Cong Q. (2020). “Sentiment analysis methods for hpv vaccines related tweets based on transfer learning,” in Healthcare (MDPI), 307. 10.3390/healthcare8030307 PMC7551482 32872330

[B65] Zhu W. Liu H. Dong Q. Xu J. Kong L. Chen J.. (2023). Multilingual machine translation with large language models: empirical results and analysis. arXiv preprint arXiv:2304.04675.

